# Prostaglandins: Key players in immunomodulation of the nervous system

**DOI:** 10.4103/NRR.NRR-D-25-00089

**Published:** 2025-09-03

**Authors:** Ziqin Wang, Ying Sun, Minjie Luo, Nina He, Zhongchi Wen, Zuzhen Wang, Yonglong Zhang, Jie Zhao, Ying Liu

**Affiliations:** 1 Department of Neurosurgery, Xiangya Hospital, Central South University, Changsha, Hunan Province, China; 2 Department of Pathophysiology, Xiangya School of Medicine, Central South University, Changsha, Hunan Province, China; 3 National Clinical Research Center for Geriatric Disorders, Xiangya Hospital, Central South University, Changsha, Hunan Province, China; 4 Sepsis Translational Medicine Key Lab of Hunan Province, Changsha, Hunan Province, China; 5 National Medicine Functional Experimental Teaching Center, Changsha, Hunan Province, China; 6 Jiangsu Key Laboratory of New Drug Research and Clinical Pharmacy, Xuzhou Medical University, Xuzhou, Jiangsu Province, China

**Keywords:** autoimmune neurological diseases, cerebrovascular destructive disease, immune cells of the nervous system, immunomodulatory, nervous system, neuralgia and chronic pain, neurodegenerative diseases, neuroinflammation, prostaglandin, prostaglandin receptor

## Abstract

As a class of bioactive substances that play important roles in a variety of physiological and pathological processes, prostaglandins have attracted increasing research attention. Published studies have identified the molecular characteristics of prostaglandins in peripheral organs, the enzymes involved in prostaglandin biosynthesis, and prostaglandin receptors. Some studies have also explored the characteristics of prostaglandins and their functions in the central nervous system. In particular, for immunomodulation, prostaglandins in the brain show a unique immunosuppressive effect, which is of great importance for maintaining brain immune homeostasis and preventing excessive immune responses. In this review, we review the biosynthesis and degradation of prostaglandins, discuss the regulatory effects of prostaglandin systems on the immune cells of the nervous system, and explore the therapeutic potential of targeting prostaglandin systems for neurological diseases, thereby providing new perspectives for the treatment of these diseases. Overall, prostaglandins play important roles in the immunomodulation of the nervous system, and the complexity of the underlying processes depends on the variety and types of specific receptors and, more importantly, the cell types prostaglandins act on. Therefore, additional in-depth studies of the specific mechanisms involving prostaglandins in immunomodulation, including the interactions among different types of prostaglandins and their roles in neuropathological conditions, are essential. The prostaglandin signaling pathway may represent another important direction for the development of new drugs for neurological diseases, and the combination of prostaglandin system targets with other therapies (such as immunomodulatory drugs and gene therapy) may yield better results.

## Introduction

The term “prostaglandins” (PGs) is used to collectively refer to multiple species of eicosanoic acid compounds with a wide range of biological activities that serve as important secretory lipid mediators in humans and animals (Funk, 2001). PGs were first identified in the 1930s, when the Swedish physiologists von Euler and Goldblatt described the presence of active substances with smooth muscle–excitatory and blood pressure–lowering activities (US and Gaddum, 1931; Bergstroem and Samuelsson, 1965). PGs are of interest because of their wide range of physiologic and pathologic functions. In the brain, as an important class of bioactive lipid mediators, PGs are involved in the regulation of physiological processes such as pain, fever, and sleep (Matsumura et al., 1994; Jang et al., 2020; Blomqvist, 2023), and they also exhibit unique immunosuppressive effects in immune response and inflammatory processes (Li et al., 2019; Sallam et al., 2022).

The immune system is a complex set of defense mechanisms in the body (Parkin and Cohen, 2001) that primarily aim to recognize and defend against foreign invaders, such as viruses (Abad and Danthi, 2020), bacteria (Wiedenheft et al., 2011), and other pathogens, and to clear the body of damaged or abnormal cells (Prata et al., 2018), such as cancer cells. Dysregulation or overactivity of the immune response can lead to autoimmune diseases, allergic reactions, rejection of transplanted organs, and other complications, highlighting the importance of maintaining the homeostasis of the immune system.

The nervous system has long been considered to be “immunologically privileged,” i.e., ignored by the immune system. However neurodegenerative diseases such as Alzheimer’s disease (AD) (McGeer and McGeer, 1995) and Parkinson’s disease (PD) (Joshi and Singh, 2018) are inflammatory, and the autoimmune etiology of inflammatory diseases of the nervous system such as myasthenia gravis (MG) (Graus and De Baets, 1993) and, to some extent, multiple sclerosis (MS) (Wootla et al., 2012) may disprove this view. The nervous and immune systems regulate each other physiologically and pathologically, and work in concert with or opposition to each other (Daëron, 2022). A special class of immune cells in the nervous system, including microglia, astrocytes, and oligodendrocytes, are primarily responsible for protecting the brain from pathogens and maintaining brain health and homeostasis. In addition to these cells, macrophages and lymphocytes, although not nerve cells, perform important immune functions in the central nervous system, especially during infection or injury. PGs exert immunosuppressive effects mainly by modulating the production and release of inflammatory mediators in these specialized immune cells, inhibiting cell activation and migration, and regulating the activation and function of immune cells (Raud, 1990; Brudvik and Taskén, 2012; Vijay et al., 2017). These functions of PGs provide new perspectives and strategies for the management of neurological disorders.

Neurological diseases pose a serious threat to human health, and the existing treatment methods are limited in effect and often accompanied by notable adverse reactions. PGs activate different receptors and play a variety of roles in the central nervous system, such as promoting or inhibiting inflammation, regulating the survival and apoptosis of nerve cells, and affecting the permeability of the blood–brain barrier. However, due to the complexity of PG biosynthesis and degradation pathways as well as the functional heterogeneity of each member in different neurological diseases, more research is required before PGs can be used for the treatment of neurological diseases.

This review aims to explore the immunomodulatory effects of PGs on the central nervous system. It systematically reviews the biosynthesis, degradation pathways, and biological functions of PGs in the nervous system. It also delves into the immune-modulation mechanisms of different types of PGs on nerve cells, along with their specific manifestations in neurological diseases, to explore the potential of PG signaling pathways as new therapeutic targets. By analyzing the relationships between the PG system and the pathogenesis of neurological diseases, this review proposes a new approach to drug development centered on PG downstream targets. It also explores the feasibility of combining these PGs with immunomodulatory drugs and gene therapies, providing theoretical foundations and innovative strategies to overcome the current limitations in the treatment of neurological diseases.

## Retrieval Strategy

This study retrieved relevant articles published in the PUBMED/MEDLINE database from January 1971 to June 2025, using the following search terms: prostaglandin system and its various subtypes, neurological diseases, including but not limited to inflammatory responses, and immune regulation. Initially, studies that were irrelevant or duplicates were excluded based on their titles and abstracts. Subsequently, the full texts were reviewed, and studies that provided clear explanations of sample size, experimental methods, and procedures were included.

## Biosynthesis, Degradation, and Biological Functions of Prostaglandins in the Nervous System

### Biosynthesis

**Figure 1:**
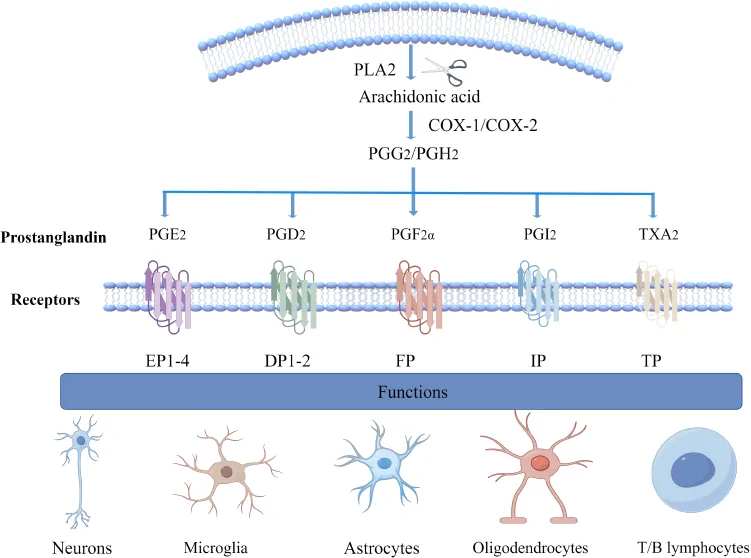
Biosynthesis and function of prostaglandins in the nervous system. When cells are stimulated, AA is released from membrane phospholipids by PLA2 and converted to PGG2 or PGH2 by COX (COX-1 and COX-2). PGG2/PGH2 are synthesized by their respective synthetases into different types of PGs (PGE2, PGD2, PGF2α, PGI2, and TXA2), these PGs exert immunomodulatory effects and various biological functions on cells of the nervous system (such as neurons, microglia, astrocytes, and oligodendrocytes) as well as on T/B lymphocytes produced in the central nervous system under specific pathological conditions by binding to their specific receptors (EP1–4, DP1–2, FP, IP, and TP). Created with Figdraw (license code: Pxrxw996b4). COX-1/-2: Cyclooxygenase-1/-2; DP1–2: prostaglandin D2 receptor 1–2; EP1–4: prostaglandin E2 receptor 1–4; FP: prostaglandin F2α receptor; IP: prostaglandin I2 receptor; PLA2: phospholipase A2; PGG2: prostaglandin G2; PGH2: prostaglandin H2; PGE2: prostaglandin E2; PGD2: prostaglandin D2; PGF2α: prostaglandin F2α; PGI2: prostaglandin I2; TXA2: thromboxane A2.

PGs are derived from arachidonic acid, a polyunsaturated fatty acid, in the cell membrane (Bergstroem et al., 1964). The biosynthesis of PGs is mainly achieved through three successive enzymatic reactions (**Figure 1**). When cells are stimulated by various factors such as injury or infection, arachidonic acid is released from membrane phospholipids by phospholipase A2 (PLA2). Once released, arachidonic acid is sequentially converted by cyclooxygenase (COX) to the PG intermediate metabolites prostaglandin G2 and prostaglandin H2 (PGH2) (Yao and Narumiya, 2019; Talabieke et al., 2025), and downstream PG terminal synthetase/isomerase finally converts these intermediates to prostaglandin E2 (PGE2), prostaglandin D2 (PGD2), prostaglandin I2 (PGI2, also known as prostacyclin), prostaglandin F2α (PGF2α), and thromboxane A2 (TXA2), among other biologically active PGs. In mammals, PGs are classified into two major groups, prostacyclopentanes and thromboxanes, among which prostacyclopentanes are named with letters A to J, i.e., prostaglandin A (PGA), prostaglandin B (PGB), prostaglandin C (PGC), prostaglandin D (PGD), prostaglandin E (PGE), prostaglandin F (PGF), prostaglandin G/H (PGG/H), prostaglandin I (PGI), and prostaglandin J (PGJ), on the basis of differences in their chemical structures; in contrast, thromboxanes consist of thromboxane A (TXA) and thromboxane B (TXB) (An et al., 2021).

In the nervous system, PGs are mainly produced by neurons, microglia activated during brain inflammation, and reactive astrocytes (Murphy et al., 1988). Activation of COX in astrocytes enables the synthesis of PGE2, which can promote neuronal survival and differentiation by activating PGE receptor 2 (EP2) (Molina-Holgado et al., 2000). In addition, Klawonn et al. (2021) showed that activation of microglia increases interleukin (IL)-6 expression in the brain, which is involved in the pathological process of depression. Their study also showed that microglia-derived PG synthesis and release reduced neuronal excitability as well as induced aversive behavior.

The biosynthetic process of PGs is tightly regulated by a series of enzymes. COX, also known as PGH synthase, is a key enzyme in the synthesis of PGs and performs the dual functions of dioxygenation and peroxidation (Smith et al., 2011; Li et al., 2018). COX, as the name suggests, is involved the creation of a ring in a fatty acid and the addition of some oxygen molecules to form the intermediate product of PGs. Two isoforms of COX, COX-1 and COX-2, have been identified to date. These isoforms are homodimers (Picot et al., 1994; Kurumbail et al., 1996; Li et al., 2018) showing functional “heterodimer” properties (Dong et al., 2011). Among them, COX-1 is a “component enzyme” that is produced in most tissues, is naturally occurring, and is mainly involved in normal physiological activities such as platelet aggregation and gastrointestinal mucosal protection. COX-1 levels are relatively stable *in vivo*, which is essential for maintaining the homeostasis of the body’s internal environment (Kargman et al., 1996; Dovizio et al., 2014; Sim et al., 2018; Mabrok and Mohamed, 2019). However, in the brain, especially in the hippocampus, COX-2 shows high basal expression, whereas *COX-1* mRNA and protein levels in the hippocampus are increased by inflammatory stimuli (Hein and O’Banion, 2009). Other enzymes involved in PG biosynthesis include hematopoietic PGD synthase (H-PGDS), lipid transporter PGD synthase, microsomal PGE synthase-1 (mPGES-1), microsomal PGE synthase-2 (mPGES-2), cytoplasmic PGE synthase (cPGES), PGF synthase, PGI synthase, and TXA synthase (Smyth et al., 2009; Ricciotti and FitzGerald, 2011; Seo and Oh, 2017). These enzymes are responsible for the synthesis of PGE2, PGD2, PGI2, PGF2α, and TXA2, respectively, which are predominantly found in mammals.

In addition to the enzymes and substances mentioned above, other factors are also involved in the synthesis of PG. A growing body of research has highlighted the intricate relationship between imbalance of the neuroimmune microenvironment and the dynamic changes in the gut microbiota. PGs, as key messengers of lipid metabolism, not only function as the core medium of inflammatory signal transduction in immune cells, but are also affected by the metabolites of intestinal microorganisms. This relationship is mediated by two key factors, the gut–brain axis and short-chain fatty acids (SCFAs). In an inflammatory state, cytokines such as the inflammatory mediators tumor necrosis factor α (TNF-α) and IL-1β can induce the expression of PG synthetase, which affects PG synthesis and exerts a pro-inflammatory effect (Lencel et al., 2011). The gut–brain axis and SCFAs can influence this process through several pathways: SCFAs can regulate the differentiation of intestinal immune cells, promote the production of anti-inflammatory regulatory T cells (Tregs), and inhibit the secretion of pro-inflammatory factors such as TNF-α and IL-1β by Th1/Th17 cells. This reduces the upstream signaling that induces PG synthesis at the source (Du et al., 2022; Gélineau et al., 2024). Additionally, SCFAs can directly act on glial cells in the central nervous system, reducing the transcription levels of enzymes like COX-2 by inhibiting the nuclear factor-κB pathway, and thereby decreasing the production of PGs (Zhou et al., 2021; Dong and Cui, 2022). PGs themselves have an inflammation-amplifying effect, which can promote the release of pro-inflammatory cytokines through feedback mechanisms to form an inflammatory cascade reaction. PGs favor the persistence of inflammation, leading to chronic inflammation, and act as cytokine amplifiers, which can trigger the release of pro-inflammatory cytokines, activate Th cells, recruit immune cells, and increase the expression of pro-inflammatory genes (Al-Kuraishy et al., 2024). The neural and humoral pathways of the gut–brain axis play crucial regulatory roles in this process. The vagus nerve transmits SCFAs to the brain, signaling the production of neurotransmitters by intestinal endocrine cells, which regulate the hypothalamic-pituitary-adrenal axis, inhibit stress-related PGs synthesis, and thereby block the inflammatory feedback loop (Wang et al., 2022). Additionally, SCFAs enhance the intestinal barrier function, reducing the entry of pathogen-related molecular patterns (such as lipopolysaccharide [LPS]) into the circulatory system, lowering systemic inflammation, and indirectly mitigating the amplification effect of PGs on inflammation (Yue et al., 2022).

Epigenetic mechanisms also participate in and regulate the synthesis of PGs through DNA methylation, histone modification, non-coding RNA regulation, and other processes. In DNA methylation, the methylation status of the promoter region can affect the expression of key enzymes for PG synthesis. Methylation usually inhibits transcription, while demethylation has the opposite effect; thus, both processes affect PG synthesis (Deng et al., 2021; Xiao et al., 2024). One study showed that deficiency of methyltransferase-like 14 in brown adipose tissue could increase the expression of PGE2, PGD2, and PGF2α, indicating that m6A methylation could regulate PG synthesis by affecting the expression of PG synthetase-related enzyme genes (Xiao et al., 2024). During histone modification, histone acetylation and methylation change the conformation of chromatin. Inflammatory factors can recruit related enzymes to change histone modification in the COX-2 promoter region and regulate its transcription. Exposure to diesel particulate matter has been shown to increase histone H4 acetylation at the COX-2 promoter, leading to the degradation of histone deacetylase 1 and the recruitment of histone acetyltransferase p300 to the *COX-2* gene promoter. Thus, histone acetylation can regulate COX-2 transcription (Cao et al., 2007). Furthermore, one study showed that palmitic acid affects the reduction of histone H3 lysine 27 *(H3K27)me3* in human periodontal ligament fibroblasts stimulated by LPS from *Porphyromonas gingivalis*. The decrease in *H3K27* trimethylation can lead to excessive inflammatory responses in cells by altering the activity of histone methyltransferases, which may be regulated through COX-2/PGE2 pathways. Thus, histone methylation modifications can regulate COX-2 expression, thereby affecting PG synthesis (Schuldt et al., 2022). At the non-coding RNA level, microRNA (miRNA) can bind to the 3′ untranslated region of mRNA molecules encoding proteins such as COX-2 to inhibit translation or promote their degradation; similarly, long non-coding RNA can regulate chromatin structure by sponging adsorption of miRNA or regulate PG synthesis by binding to protein. An miRNA-26a mimic was shown to significantly reduce the level of COX-2 protein and inhibit the production of pro-inflammatory cytokines in macrophages stimulated by LPS. Moreover, a double-luciferase reporter gene experiment confirmed that miRNA-26a shows a targeted binding relationship with the 3′ untranslated region of *COX-2* mRNA (Yu et al., 2022). COX-2 antisense RNA 1 (PACER) is a long non-coding RNA located upstream of the COX-2 promoter region. In colorectal cancer, PACER upregulates COX-2 and PGE2 expression through the nuclear factor-κB pathway, promoting tumor cell proliferation and invasion. Knocking down PACER can reduce COX-2 and PGE2 mRNA and protein levels. Luciferase assays showed that PACER can regulate the COX-2 promoter region (Sun et al., 2021b).

Peroxisome proliferator-activated receptors are a class of nuclear receptors that can be activated by fatty acid derivatives and PGs and are involved in the regulation of inflammatory responses and PG biosynthesis (Kirkby et al., 2020). In addition, polyunsaturated fatty acid-rich fish and nuts, among other foods, can promote PG production by providing precursors for PG synthesis (Abayasekara and Wathes, 1999). Interestingly, antioxidants and phytochemicals in food can also indirectly influence PG biosynthesis by inhibiting the production of inflammatory mediators (Costea et al., 2022).

### Degradation

Much like their biosynthesis, degradation of PGs is also achieved through the regulation of a series of enzymes. The most important of these are the 15-hydroxyprostaglandin dehydrogenases (15-PGDHs). 15-PGDHs consist of type I NAD^+^-dependent 15-PGDH and type II NADP-dependent 15-PGDH (Sun et al., 2021a). 15-PGDHs have high substrate specificity and act mainly on the hydroxyl group at the C15 position of PGs to oxidize them to keto radicals, thereby significantly reducing the biological activity of PGs (Tai et al., 2006). These enzymes have been purified and cloned in different mammals (e.g., humans, rats, mice, and cows) and are widely distributed in mammalian tissues (Ma et al., 2014). The expression of 15-PGDH in neurons plays an important role in neuronal survival and protection, which was confirmed by Xu et al. (2024). Overexpression of 15-PGDH in brain tissue or primary cultured neurons has been shown to significantly exacerbate brain injury and neuronal cell death in ischemic stroke, and inhibition of 15-PGDH was shown to significantly prevent these neurological injuries (Xu et al., 2024). 15-PGDH, together with COX, controls the cellular levels of PGs, and their reciprocal regulation within the same cell appears to determine cell fate (Tai et al., 2006). Metabolic enzymes related to the degradation of PGs also involve members of the aldo-keto reductase family and cytochrome P450 monooxygenases (Bishop-Bailey et al., 2014; Jara-Gutiérrez and Baladrón, 2021; Satoshi et al., 2021). In addition, fatty acid chain shortening through lipid oxidation and β-oxidation is another pathway of PG degradation, and cyclopentenone PGs are lipid oxidation products of PGD2 (Masuda et al., 2006).

In the nervous system, neurons are not only recognized as potential producers of PGs, but are also involved in the degradation of PGs through the expression of 15-PGDH. Corwin et al. (2018) found that 15-PGDH expression was upregulated in the neurons of rats by microinfusion of PGJ2, which is derived from PGD2. Astrocytes, the main support cells in the central nervous system, have also been shown to express 15-PGDH, and a significant increase in PGE2 levels in amyotrophic lateral sclerosis (ALS) model mice has been attributed to an imbalance in the 15-PGDH-dependent clearance system (Miyagishi et al., 2017). Microglia are resident immune cells in the central nervous system. Cossarizza et al. (2019) reported that microglia activated in response to inflammation or injury can also express 15-PGDH to accelerate PG clearance, thereby limiting inflammation and maintaining PG homeostasis in the nervous system. Corwin et al. (2018) also detected 15-PGDH in oligodendrocytes, although the exact mechanism underlying this finding remains unclear. In summary, the synthesis and degradation of PGs in the nervous system is a highly regulated process involving the interaction of multiple cell types and enzymes, and these interactions are crucial for the regulation of PGs in the nervous system.

### Biological functions

In the nervous system, PGs are widely distributed in all forms of neural tissue, and different bioactive PGs play their biological roles by binding to their specific receptors. All PG receptors have been cloned, including PGD receptor 1 (DP1), PGD receptor 2 (DP2), PGE receptor 1 (EP1), EP2, PGE receptor 3 (EP3), PGE receptor 4 (EP4), PGF receptor (FP), PGI receptor (IP), and TXA2 receptor (TP), which belong to the transmembrane G protein-coupled receptor (GPCR) family (Zeng et al., 2023). These receptors are mainly involved in the regulation of inflammation, neuroprotection, release of neurotransmitters and neuronal activity, sleep regulation, pain perception, vasoregulation, learning and memory, development and maturation of the nervous system, neuroendocrine interactions, and other processes (**Table 1**). In addition, endothelial dysfunction in PG-mediated blood–brain barrier permeability and neuroinflammation also play key roles. PGE2 activates the cyclic adenosine monophosphate (cAMP)/protein kinase A (PKA) pathway through EP2/EP4, significantly increasing blood–brain barrier permeability (Khan et al., 2019). Moreover, in an experimental model of transient focal cerebral ischemia, ischemic treatment with the EP1 antagonist SC-51089 or *EP1* gene deletion resulted in significantly reduced blood–brain barrier damage and reduced hemorrhagic transformation. These neurovascular protective effects mediated by EP1 inactivation were associated with a significant reduction in the expression of matrix metalloproteinase-9/-3, reduction in peripheral neutrophil infiltration, and retention of zona occludens-1, a tight junction protein that constitutes the blood–brain barrier (Frankowski et al., 2015). PGE2 can affect the transport process related to multidrug resistance-associated protein 4, and thereby influence the functioning of the blood–brain barrier (Akanuma et al., 2010). Consequently, the effects of PGE2 on blood–brain barrier-related proteins can be achieved by affecting their transport proteins.

**Table 1:** Biological functions of PGs in the nervous system

PG	Receptor	Function	Description	Reference
PGE2, PGF2α	EP2, EP4, FP	Modulation of inflammation	Affected the activity of microglia and astrocytes and regulated the production of inflammatory mediators.	Mohan et al., 2018; Ji et al., 2021
PGE2, PGI2	EP1, EP4, DP1	Neuroprotection	Protected neurons from further damage in brain injury or neurodegenerative diseases.	Pradhan et al., 2017; Xu et al., 2022b
PGE2, PGF2α	EP2, FP	Neurotransmitter release	Affected the release of neurotransmitters and neuronal activity, such as glutamate, gamma-aminobutyric acid, and dopamine.	Arai et al., 2003; Clasadonte et al., 2011
PGD2	DP1	Sleep regulation	PGD2 is the most potent endogenous sleep-promoting substance and promotes sleep by activating PGD2 receptors.	Ahmad et al., 2019
PGI2, PGE2	IP, EP1	Pain perception	Prostaglandins such as PGE2 stimulate nociceptors and enhance pain signaling by activating G protein-coupled receptors EP1-EP4.	Murata et al., 1997; Stock et al., 2001
PGE2, PGD2, PGI2, TXA2	EP4, DP1, IP, TP	Angiogenesis	Influenced cerebral vasodilation and contraction and regulates cerebral blood flow.	Assumpção et al., 2010; Zhang and Daaka, 2011; Ahmad, 2014
PGE2	EP3	Learning and memory	Involved in learning and memory processes that affect cognitive function.	Hein and O'Banion, 2009
PGE2	EP2	Development and maturation of the nervous system	Involved in neuronal migration, synapse formation and synaptic pruning.	Sang et al., 2005; Jung et al., 2021
PGE2	EP3	Neuroendocrine interactions	Influenced the endocrine system through the hypothalamic-pituitary-adrenal axis and regulates the immune response.	Elander et al., 2009; Zhang et al., 2011

DP1: Prostaglandin D2 receptor 1; DP2: prostaglandin D2 receptor 2; EP1: prostaglandin E2 receptor 1; EP2: prostaglandin E2 receptor 2; EP3: prostaglandin E2 receptor 3; EP4: prostaglandin E2 receptor 4; FP: prostaglandin F2α receptor; IP: prostaglandin I2 receptor; PG: prostaglandin; PGE2: prostaglandin E2; PGD2: prostaglandin D2; PGF2α: prostaglandin F2α; PGI2: prostaglandin I2; TP: thromboxane A2 receptor; TXA2: thromboxane A2.

## Immunomodulatory Effects of Prostaglandins on the Nervous System

### PGE2

PGE2 is one of the most abundantly produced PGs in the body and can be produced by almost all cell types in the body, including epithelial cells, fibroblasts, and, especially, infiltrating inflammatory cells (Cheng et al., 2021; Li et al., 2025). PGE2 is mainly produced by three previously identified and characterized PGE synthases, namely, mPGES-1 (Tanioka et al., 2000), microsomal PGE synthase-2 (mPGES-2) (Jakobsson et al., 1999), and cPGES, which is responsible for the production of PGE2 from PGH2 (Tanikawa et al., 2002). The constitutive cPGES is mainly coupled to COX-1 (Tanioka et al., 2000), whereas the inducible mPGES-1, which is stimulated by inflammatory factors, is mainly coupled to COX-2 and increases PGE2 production. The expression of mPGES-2 and cPGES is constitutive rather than regulatory. In contrast to mPGES-1 and cPGES, mPGES-2 shows no subspecific preference for PGE2 production. mPGES-1 is usually associated with specific types of inflammation and pain, whereas mPGES-2 is involved in PGE2 production in a number of other physiological and pathological conditions (Murakami et al., 2003). To perform the subsequent functions, synthesized PGE2 can be actively transported by the PG efflux transporter protein multidrug resistance protein 4 in addition to being excreted from the cells by simple diffusion (Reid et al., 2003; Cheng et al., 2021).

**Figure 2:**
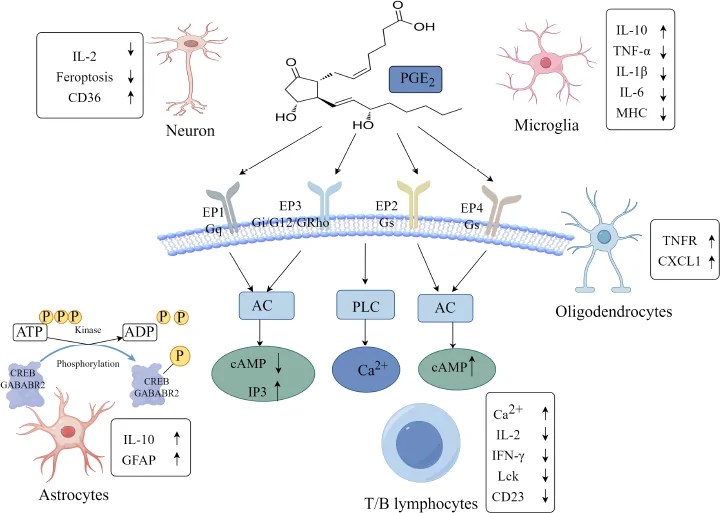
The mechanism of PGE2 immunosuppression in the nervous system. PGE2 mediates downstream signaling pathways by activating four different G protein-coupled receptors (EP1-4), EP1 binds to Gq, EP3 binds to Gi, inhibits AC and inhibits the formation of cAMP; EP2 and EP4 bind to Gs and stimulate AC to increase the production of cAMP in cells; in addition, the activation of PLC can lead to an increase in intracellular calcium concentration. After binding to specific receptors on the cell membrane, PGE2 mainly exerts its immunosuppressive effect on neurons, microglia, astrocytes, oligodendrocytes and T/B lymphocytes in the central nervous system. PGE2 indirectly affects neuronal activity by inhibiting IL-2 expression and iron death, inhibiting the production of pro-inflammatory cytokines (TNF-α, IL-1β, and IL-6), inhibiting activation of microglia and T/B lymphocytes, and inhibiting the expression of MHC on microglia, limiting antigen presentation and facilitating the transition to an anti-inflammatory M2 phenotype. PGE2 can also regulate the immune function of astrocytes by affecting the phosphorylation status of CREB, and GABABR2D. Created with Figdraw (license code: z=weS6b116). AC: Adenylyl cyclase; cAMP: cyclic adenosine monophosphate; CREB: cAMP response element-binding protein; CXCL1: C–X–C chemokine ligand 1; EP1–4: prostaglandin E receptor 1–4; GFAP: glial fibrillary acidic protein; GABABR2: gamma-aminobutyric acid type B receptor 2; Gq: G protein subunit q; Gs: G protein subunit s; GRho: Rho family G protein; G12/13: G protein subunit 12/13; IFN-γ: interferon-gamma; IL-2/-6/-10: interleukin-2/-6/-10; IP3: inositol trisphosphate; LCK: lymphocyte-specific protein tyrosine kinase; MHC: major histocompatibility complex; PLC: phospholipase C; PGE2: prostaglandin E2; TNF-α: tumor necrosis factor-alpha; TNFR: tumor necrosis factor receptor.

PGE2 is the major primary PG produced by brain cells (Vazquez-Tello et al., 2004) and mediates downstream signaling pathways through the activation of four different GPCRs (EP1–4). EP1 is located on human chromosome 19 at locus p13.12 and contains two introns and three exons encoding GPCRs (Funk et al., 1993). EP1 is coupled to Gq and mimics phospholipase C. Phospholipase C cleaves phosphatidylinositol diphosphate (PIP2), generating inositol trisphosphate (IP3) and diacylglycerol. IP3 binds to the IP3 receptor in the endoplasmic reticulum, releasing calcium from the endoplasmic reticulum and leading to an increase in the intracellular concentration of calcium. Diacylglycerol activates calcium-dependent protein kinases (PKCs) (Tang et al., 2005). EP2 is located on human chromosome 14 at position p22.1 and contains two introns and three exons encoding GPCRs (Regan et al., 1994). EP3 and EP4, on the other hand, are located on human chromosome 1 at position p31.1 and on human chromosome 5 at position p13.1, respectively. EP4 contains seven exons encoding GPCRs of the retinoid-like receptor family (Bastien et al., 1994; Kotani et al., 1997). Binding of EP2 and EP4 to Gs stimulates adenylyl cyclase and increases intracellular cAMP (Sagana et al., 2009; Yagami et al., 2016). EP3 binds to Gi, inhibits adenylyl cyclase, and suppresses cAMP formation (Yagami et al., 2016; **Figure 2**).

Neurons, the basic structural and functional unit of the nervous system, consist of three parts: cytosol, dendrites, and the axon (Sun et al., 2025). Neurons have several notable characteristics such as excitability, conductivity, plasticity, and secretion (Naffaa, 2025). PGE2 can affect the excitability of neurons by modifying their electrical activity through the regulation of ion channels (potassium channels) (Primi and Bueno, 1986; Lei et al., 2012) and N-methyl-D-aspartate (NMDA) receptors (Hou and Zhang, 2017). The PGE2 signaling pathway plays an important role in neuronal inflammatory responses. Studies using a model of sustained status epilepticus showed that significantly elevated levels of PGE2 promoted the production of inflammatory factors in neuronal cells and reactive gliosis (e.g., proliferation and hypertrophy of microglia and astrocytes) (Ryley Parrish et al., 2013; Rojas et al., 2019a). Thus, PGE2 may exacerbate the inflammatory response of neurons by increasing the release of inflammatory factors. However, PGE2 synthase inhibitors can significantly reduce the inflammatory response in neurons, as evidenced by the reduced mRNA expression of pro-inflammatory and chemokine factors as well as the decreased protein levels of glial biomarkers (Lee et al., 2019). Thus, PGE2 may promote neuronal inflammatory responses, and inhibition of its synthesis attenuates such inflammatory responses. PGE2 can also regulate neuronal metabolism and function through its receptors, such as EP2. During aging, activation of the PGE2-EP2 signaling pathway leads to an increase in the conversion of glucose to glycogen and a decrease in glucose utilization, limiting mitochondrial respiration (Chen et al., 2022). This metabolic change may affect neuronal energy supply and function. Inhibition of the PGE2/EP2 signaling pathway restores neuronal metabolic capacity and improves glucose utilization and mitochondrial function, thereby improving neuronal health. The anti-inflammatory or inflammation-inhibiting effects of PGE2 on neurons are dependent on its downstream activated receptors. Ablation of EP2 or EP3, which are PGE2 receptors, has been shown to attenuate inflammatory responses, amyloid accumulation, and synaptic protein loss in familial AD models (Liang et al., 2005b; Hoshino et al., 2007; Shi et al., 2012; Johansson et al., 2015). In primary cultured neurons undergoing oxygen-glucose deprivation/reperfusion, EP3 and EP4 expression is increased (Xu et al., 2021) and can exert neuroprotective effects by inhibiting iron death (Xu et al., 2022b). However, EP1 was shown to cause neurotoxic effects in a model of cerebral ischemia (Kawano et al., 2006). In addition, PGE2 indirectly inhibits neuronal immune responses by affecting the activation and function of immune cells. PGE2 reduces the antigen-presenting function of monocyte macrophages and the production of IL-2, thereby inhibiting the activation and proliferation of T cells and natural killer cells (Valitutti et al., 1989; Faist et al., 1996). These immune cells have protective and reparative effects on neurons within the central nervous system, so the immunosuppressive effects of PGE2 may indirectly affect neuronal health.

Microglia are a class of non-neuronal cells in the central nervous system, including the brain and spinal cord, that consists mainly of microglia and their precursor cells (Aarum et al., 2003). Together with neurons, they constitute the major cellular components of the brain and spinal cord (Bellver-Landete et al., 2019) and are involved in maintaining and supporting neuronal function (Zha et al., 2025). Microglia play important roles in regulating the external environment of neurons, metabolic waste removal, and neuronal regeneration and repair (Damisah et al., 2020). They are also involved in immune responses and neuroinflammatory processes (Zhang et al., 2024). PGE2 has been shown to inhibit TNF-α production and promote IL-10 production (Strassmann et al., 1994). Recent studies have highlighted the relevance of microglial innate immune activation in neurodegenerative diseases such as AD (Leng and Edison, 2021) and PD (Liu et al., 2022). However, microglial activation is a double-edged sword: on one hand, it counteracts neurotoxicity by phagocytosis of amyloid-β (Aβ), but on the other hand, it activates the innate immunity system, causing paracrine damage to neurons. Knockdown of microglial EP2 enhances Aβ phagocytosis without paracrine neurotoxicity (Shie et al., 2005). Primary mouse microglia lacking the PGE2 receptor EP2 showed reduced innate immune-mediated neurotoxicity and increased phagocytosis of Aβ peptides (Jin et al., 2007). A subsequent study demonstrated that innate immune activation in the hippocampus of wild-type mice suppressed CD36 expression through an EP2-dependent mechanism (Li et al., 2015). Recent studies have also indicated that PGE2 plays an important role in neuroinflammation signaling through EP2, since genetic ablation or pharmacological inhibition of EP2 reduced the induction of large amounts of many key pro-inflammatory mediators, including the release of the cytokines IL-1β, IL-6, and TNF-α by activated microglial cells (Andreasson, 2010; Johansson et al., 2013; Quan et al., 2013; Rojas et al., 2019b). PGE2 also promotes the production of anti-inflammatory cytokines such as IL-10 by microglia through the activation of EP2 and EP4; these cytokines inhibit the production of pro-inflammatory cytokines and thus reduce the inflammatory response. However, injury (e.g., pathogens or tissue damage) can induce increased production of pro-inflammatory cytokines such as IL-1β or TNF-α in microglia and enhance immune responses, including cell recruitment through chemotaxis and phagocytosis (Subhramanyam et al., 2019; Leng and Edison, 2021). Microglia not only act through EP2 but also express EP1 (Shie et al., 2005). In particular, with activation of EP1, high levels of PGE2 may exacerbate the inflammatory response by increasing the production of pro-inflammatory cytokines. Singh et al. (2013) demonstrated that EP1 is expressed in activated microglia and contributes to microglial activation, and that amyloid plaques in EP1-deficient AD mice are reduced in comparison with those in control AD mice; moreover, Aβ-induced toxicity and Ca^2+^ responses were also reduced in EP1^–/–^ neurons than in control neurons. Microglia can show a classical pro-inflammatory state (M1) or an alternative anti-inflammatory state (M2) (Wang et al., 2018), and PGE2 can mediate the transformation from the pro-inflammatory to anti-inflammatory state. In addition, PGE2 can limit antigen presentation by inhibiting the expression of the major histocompatibility complex on microglia, thereby impairing cytotoxic T-cell responses, and can also increase the proportion of Tregs, which suppress the immune system in brain tumors (Toki et al., 2015).

In the nervous system, astrocytes are also considered an important immune cell population. Cultured astrocytes have been shown to resemble reactive astrocytes (Lee et al., 1999), due to the fact that treatment with PGE2 increases the level of glial fibrillary acidic protein and decreases the reactivity of Aβ mRNA in cultured astrocytes (Lee et al., 1997). Astrocytes have recently been categorized as “A1” and “A2” on the basis of their molecular markers (Liddelow et al., 2017). Reactive astrocytes expressing “A1” markers have been identified in a variety of adult neurodegenerative diseases and are thought to have neurotoxic effects (Pérez-Núñez et al., 2025). In a transcriptional assessment of reactive astrocyte subtypes in a mouse model, induction of COX-2 was associated with the “A2” phenotype (Zamanian et al., 2012). Although astrocytes are mainly considered to be neuronal support cells, they also play key roles in inflammatory and immune responses. Astrocyte membranes can express a variety of pattern recognition receptors that interact with damage-associated molecular patterns to trigger inflammation (Farina et al., 2007). The damage-associated molecular patterns released by necrotic cells or secreted by inflammatory cells can activate the COX-2/PGE2 astrocyte signaling pathway, promoting various types of central nervous system injuries (Zhang et al., 2019). Astrocytes also express several Toll-like receptors and establish a response to innate immune triggers by releasing pro-inflammatory molecules (Gorina et al., 2011). On the other hand, they can also limit the spread of inflammatory cytokines or injury-induced toxic molecules through physical glial scarring and/or secretion of anti-inflammatory cytokines. For example, the anti-inflammatory cytokine IL-10 can be released from human astrocyte lines stimulated by interferon (IFN)-γ1a or a combination of pro-inflammatory cytokines (LPS/IFN-γ) plus IFN-γ1a (Mohsenzadegan et al., 2015). PGE2 has been shown to induce phosphorylation of cyclic phosphoadenylate response element-binding protein (CREB) and γ-aminobutyric acid B receptor 2 (GABABR2) in cultured rat cerebral cortical astrocytes, and the phosphorylation levels of CREB and GABABR2 in astrocytes have been shown to be significantly elevated in a time-dependent manner after PGE2 stimulation. Thus, PGE2 may regulate the immune function of astrocytes by affecting the phosphorylation status of these molecules (Lin et al., 2015).

Oligodendrocytes are highly specialized cells of the central nervous system that are produced by oligodendrocyte progenitor cells (OPCs) (Bradl and Lassmann, 2010). Oligodendrocytes are primarily involved in the formation of myelin, a multilayered fatty material that wraps around axons to ensure their insulation and facilitate the conduction of nerve impulses. In addition to their role in myelin production and renewal, OPCs also have the ability to control tissue homeostasis and to sense and respond to inflammation (Boccazzi et al., 2022). In experimental models of central nervous system injury, deficiency of OPCs has been shown to exacerbate neuroinflammation and neurodegenerative pathologies. In mice that underwent ablation of neural glial antigen 2, microglia showed aberrant activation, leading to a more aggressive LPS-induced neuroinflammatory response (Skripuletz et al., 2011). However, administration of mouse recombinant hepatocyte growth factor in OPC-ablated rats afforded significant protection of hippocampal neurons and attenuated microglial activation (Nakano et al., 2017). Another study suggested that TNFR2 activation on oligodendrocytes may inhibit inflammatory signaling and limit excessive neuroinflammation (Madsen et al., 2020). PGE2 (Carlson et al., 2015), platelet-derived growth factor (Redwine et al., 1997) and hepatocyte growth factor (Yan and Rivkees, 2002), the chemotactic molecules netrins and secreted signaling elements (Tsai et al., 2003), and the chemokine C–X–C chemokine ligand 1 (CXCL1) (de Castro and Bribián, 2005) appear to be involved in the maturation of OPCs to oligodendrocytes. Production of COX-2/PGE2 by reactive astrocytes has been shown to inhibit the maturation of oligodendrocytes in neonatal white matter injury, which is dependent on the PGE2 receptor EP1 (Shiow et al., 2017). The other receptor EP2 regulates cell differentiation through specific activation of Wnt pathway signaling (Castellone et al., 2005; Goessling et al., 2009) and can cause maturation arrest in OPCs (Fancy et al., 2009). In contrast to these results, activation of EP3 in OPCs may be associated with changes in cAMP levels or an increase in intracellular calcium levels (Carlson et al., 2015). Moreover, COX-2, a rate-limiting enzyme for PGE2 synthesis, has been shown to be involved in neuroexcitotoxicity (Smith et al., 1996; Kelley et al., 1999), and inhibition of COX-2 is attributed to an immunomodulatory effect resulting from the inhibition of T-cell signaling via IL-12 (Muthian et al., 2006).

T/B lymphocytes are a part of the adaptive immune system and are not usually present in large numbers in the central nervous system, except under specific pathological conditions such as autoimmune diseases or MS (Zafranskaya et al., 2013). The central nervous system of aged mice shows abnormal expression of CD4^+^ T cells, CD8^+^ T cells, B cells, and myeloid cells (Mestre et al., 2021). The immunosuppressive effects of PGE2 on T/B cells are mainly mediated through the activation and proliferation of T/B cells (Smith et al., 1971a, b; Prijatelj et al., 2011). cAMP, T-cell receptor (TCR)/B-cell receptor, and PKA may be important factors for regulation. One study identified potential inhibitory pathways of PGE2 in T cells, mainly by affecting the production of cAMP, which exerts its antiproliferative effects by interfering with IL-2-mediated gene expression and eliminating the TCR-mediated increase in cytoplasmic Ca^2+^ (Wehbi and Taskén, 2016). PGE2 may also inhibit IL-2 and IFN-γ production in Th1, but not IL-4 and IL-5 production in Th2, by altering the polarization of T helper cells toward Th1 and Th2 subtypes (Alder et al., 2006). Lymphocyte cell-specific protein tyrosine kinase (Lck) is responsible for TCR signaling following antigen recognition, and inhibition of Lck has also been suggested to inhibit PGE2 triggered T-cell activation (Chemnitz et al., 2006). In addition, PGE2 activates hematopoietic progenitor kinase 1 activity through a PKA-dependent pathway, a unique signaling pathway that allows PGE2 to bind to negative regulators of the TCR signaling pathway and use them to inhibit T-cell activation (Sawasdikosol et al., 2007). In B cells, PGE2 mainly inhibits the proliferation and differentiation of immature B cells, whereas it shows a significant proliferation-promoting effect on mature B cells. PGE2 inhibits IL-4 induced Fc epsilon R2/CD23 expression on lymphoid B cells by increasing intracellular cAMP levels (Galizzi et al., 1988) and can promote B-cell receptor-induced apoptosis of B-cell precursors, an apoptotic pathway that is dependent on the receptor for PGE2, EP2, and requires increased intracellular cyclic adenosine 3′,5′-monophosphate, PKA activation, and protein synthesis to inhibit B-cell activation and proliferation (Roper and Phipps, 1992; Shimozato and Kincade, 1999; Prijatelj et al., 2011).

### Prostaglandin D2

**Figure 3:**
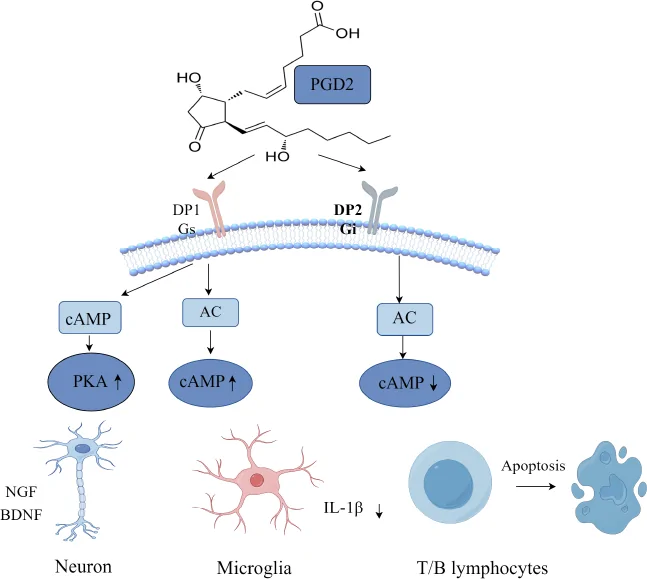
The mechanism of PGD2 immunosuppression in the nervous system. PGD2 is the most abundant prostaglandin in mouse brains, and the signaling of PGD2 cannot be simply classified as anti-inflammatory or pro-inflammatory. Normally, PGD2 can bind to DP1 at nerve endings and affect neuronal function through the PKA signaling pathway, stimulating DP1 coupling with Gs protein to increase intracellular cAMP level; and stimulating DP2 coupling with Gi protein to reduce intracellular cAMP level. Besides, PGD2 protects against brain injury by promoting neuronal expression of NGF and BDNF, enhancing antigen presentation, and promoting T-cell apoptosis, but PGD2 drives disease progression in many cases, depending on the type of receptor and the extent of brain injury. Created with Figdraw (license code: xA=q=899a5). AC: Adenylyl cyclase; BDNF: brain-derived neurotrophic factor; cAMP: cyclic adenosine monophosphate; DP1: prostaglandin D2 receptor 1; DP2: prostaglandin D2 receptor 2; Gi: G protein subunit i; IL-1β: interleukin-1 beta; NGF: nerve growth factor; PGD2: prostaglandin D2; PKA: protein kinase A.

PGD2 was previously found to be the major PG produced in the brains of various mammals, including humans (Narumiya et al., 1982; Ogorochi et al., 1984). Two synthases, lipid transporter PGD synthase or H-PGDS, evolved from different origins to fulfill the same role, i.e., the conversion of PGH2 to PGD2. Lipid transporter PGD synthase is a member of the family of lipid-transporting proteins (Narumiya et al., 1982), and is expressed primarily in the central nervous system. H-PGDS was the first identified invertebrate direct homologue of the sigma class of glutathione S-transferases (Kanaoka et al., 1997). *H-PGDS* mRNA is expressed in microglia and cerebellum during early postnatal development (Mohri et al., 2003). Once synthesized, and in the presence of serum albumin, PGD2 is metabolized rapidly and in a non-enzymatic manner to 15-deoxy-∆12, 14-pgj_2_ (15dPGJ_2_) or ∆12-pgj2, depending on the presence of serum albumin. PGD2 stimulates two different types of G-protein-coupled receptors DP1 (which couples to Gs proteins to increase intracellular cAMP levels) (Hirata et al., 1994) and DP2 (which couples to Gi proteins to decrease intracellular cAMP levels). DP2, also known as chemoattractant receptor-homologous molecule expressed on Th2 cells (CRTH2), is a chemotactic receptor homodimer expressed on the Th2 homologous molecule of chemotactic receptors (Nagata et al., 1999; Hirai et al., 2001). DP1 is involved in the regulation of sleep, pain, and food intake (Eguchi et al., 1999; Qu et al., 2006; Ohinata et al., 2008), DP2 is involved in myelin formation, adipocyte differentiation, and inhibition of hair follicle neogenesis in the peripheral nervous system (Nelson et al., 2013; Trimarco et al., 2014; Wakai et al., 2017; **Figure 3**).

In neurons, PGD2 can modulate neuronal excitability by lowering neuronal action potential thresholds. For example, PGD2 released by mast cells can bind to DP1 in nerve endings and influence neuronal function through the PKA signaling pathway (Feng et al., 2006); furthermore, sleep deprivation increases PGD2 levels in the brain, which is associated with immune collapse and the development of cytokine storms. In sleep-deprived mice, an increase in brain-derived PGD2 induces aggregation of circulating neutrophils and cytokine storm syndrome, leading to multiple organ dysfunction syndrome and death (Sang et al., 2023). This suggests that PGD2 can be involved in immune regulation through neuronal function. In general, DP1 plays a protective role in models of excitotoxicity and ischemic brain injury, whereas DP2 accelerates the process of brain injury. PGD2 exerts neuroprotective effects by significantly increasing the levels of neuronal growth factor and brain-derived neurotrophic factor (Matsugi et al., 1995). Compared with C57 mice, C57 DP1^–/–^ mice showed an increased susceptibility to ischemia-reperfusion injury. The increased susceptibility and significantly enlarged infarct area in C57 DP1^–/–^ mice compared with C57 mice suggest that DP1 plays a significant protective role in cerebral ischemia–reperfusion injury (Gelir et al., 2005). However, it has also been reported that PGD2 induces apoptosis in hippocampal neurons (Liang et al., 2005a), that DP2 exhibits high expression and excitotoxicity in normal hippocampal pyramidal neurons, and that DP2 agonists significantly augmented glutamatergic toxicity on hippocampal CA1 and CA3 neurons (Ma et al., 2016). These findings reveal that the paradoxical effects of PGD2 on neuronal effects may be related to DP type and degree of injury.

PGD2 is the most abundant PG in the mouse brain and normally mediates anti-inflammatory effects when signaled through DP1 receptors on myeloid cells (Hirai et al., 2001; Vijay et al., 2017), and the overall ablation of DP1 receptors leads to a dysregulated immune response following infection with the neuroleptic JHM strain of mouse hepatitis virus, characterized by a delayed IFN-Ι response accompanied by an exaggerated IL-1β response. This dysregulated immune response appears to be caused primarily by microglia-specific effects and results in higher viral loads and delayed viral clearance (Vijay et al., 2017). In addition, DP1 signaling has an anti-inflammatory effect in experimental autoimmune encephalomyelitis (EAE) mice. In the absence of DP1 signaling, disease incidence is lower. In this case, the absence of DP1 in dendritic cells is essential for antigen presentation, leading to enhanced activation and antigen presentation in lymph nodes. This leads to T cell apoptosis and fewer cells enter the central nervous system (Zheng et al., 2020). Notably, elevated levels of *DP1* mRNA were detected in the vicinity of plaques in patients with AD compared to normal controls (Mohri et al., 2007). *DP1* mRNA is expressed predominantly by microglia and astrocytes. Examination of Tg2576 transgenic mice that developed mouse AD showed that DP1 protein was expressed similarly to that in the vicinity of plaques, which is consistent with data from human studies (Mohri et al., 2007). In hypoxia-induced encephalitis mice, DP1 signaling is deleterious and, in addition, hematopoietic PG synthase, which is responsible for PGD2 production, is increased in microglial cells at necropsy in the brains of newborns dying from hypoxia-induced encephalitis compared to newborns dying from non-neurological causes (Taniguchi et al., 2007). Convulsive mice (lacking GalC expression, expressed by oligodendrocytes) are highlighted by spastic states, convulsions, demyelination and astrocyte proliferation. Treatment of these mice with hematopoietic PG synthase inhibitors or crossbreeding twitchy mice with DP1^–/–^ reduced clinical disease and pathological changes (Mohri et al., 2006). However, DP2 receptors in microglia are virtually absent or expressed at very low levels (Corwin et al., 2018). This suggests that DP1 signaling does not have an anti-inflammatory effect but contributes to the pathogenesis of the disease. Altogether, these results suggest that PGD2 signaling actions cannot be easily classified as anti-inflammatory or pro-inflammatory.

### Prostaglandin F2α

**Figure 4:**
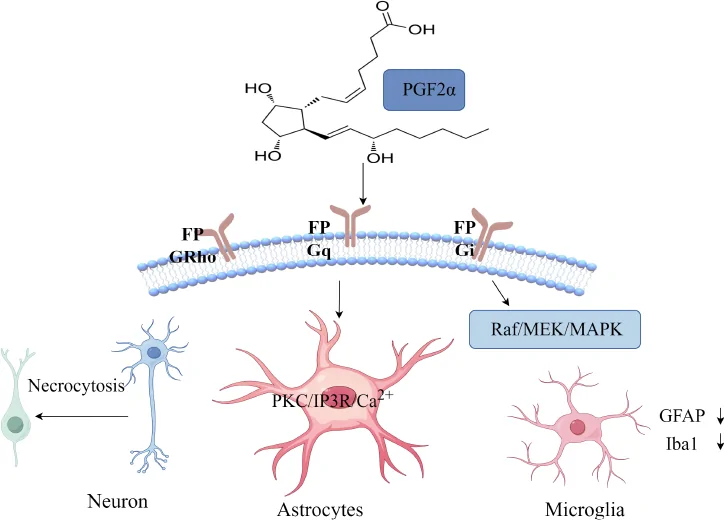
The mechanism of PGF2α immunosuppression in the nervous system. PGF2α immunomodulates the nervous system through activation of FP receptors and leads to the activation of several signaling pathways, including PKC/IP3R/Ca^2+^ and Raf/MEK/MAPK. Supraphysiological levels of PGF2α may cause neuronal necrosis. Its receptor FP may also be involved in microglia activation and astrocyte proliferation. Created with Figdraw (license code: rwSx=dda2d). FP: Prostaglandin F2α receptor; GFAP: glial fibrillary acidic protein; GRho: Rho family G protein; Gq: G protein subunit q; Gi: G protein subunit i; Iba1: ionized calcium-binding adapter molecule 1; IP3R: inositol trisphosphate receptor; MAPK: mitogen-activated protein kinase; MEK: mitogen-activated protein kinase kinase; PGF2α: prostaglandin F2α; PKC: protein kinase C.

PGF2α is present in almost all tissues, and three types of PGF synthase (PGH 9,11-endoperoxide reductase, PGD 11-ketoreductase, and PGE 9-ketoreductase) catalyze the formation of PGF2α from PGH2, PGD2, and PGE2, respectively. PGF2α usually binds to the receptor FP to fulfill various roles. The two splice variants of the FP receptor are known as FPA and FPB and are distinguished by the different lengths of the C-terminal tails (Ricciotti and FitzGerald, 2011). FP induces intracellular inositol phosphate production mainly by coupling to Gq, which in turn activates PKC by promoting Ca^2+^ release from the endoplasmic reticulum, as well as the activation of Rho via G12/G13 and the rapidly accelerated fibrosarcoma (Raf)/mitogen-activated protein kinase/mitogen-activated protein kinase pathway via Gi (Wu et al., 2023; **Figure 4**). In regulating inflammation, allergic responses, intraocular pressure and blood pressure, making it an important target for research and among the first-line drugs for the treatment of glaucoma.

Brain injury promotes the up-regulation of PGs, particularly pro-inflammatory PGF2α, and the over-activation of their cognate G-protein coupled FP receptors, which may exacerbate neuronal damage (Glushakov et al., 2013). The concentration of PGF2α is elevated in the cerebrospinal fluid of participants with progressive MS, and increases as the disease progresses (No authors listed, 1981). The FP coupled to Gq is expressed predominantly in astrocytes, but not in neurons and does not directly affect neuronal survival in neuron-rich cultures (Glushakov et al., 2013). Activation of FP receptors triggers a variety of events including stimulation of the PKC/inositol 1,4,5-triphosphate receptor/Ca^2+^ signaling pathway, through which supraphysiological levels of PGF2α may lead to intracellular calcium overload, which can result in the excessive release of excitatory neurotransmitters, and may even trigger neuronal necrosis. In addition, FP receptors may be involved in microglia activation and astrocyte proliferation. In one study, treatment with the selective FP antagonist AL-8810 significantly reduced CCI-induced hippocampal swelling as well as levels of markers of gliosis and microglia activation (i.e., glial fibrillary acidic protein and ionized calcium-binding adapter molecule 1) (Glushakov et al., 2013).

### Prostaglandin I2

**Figure 5:**
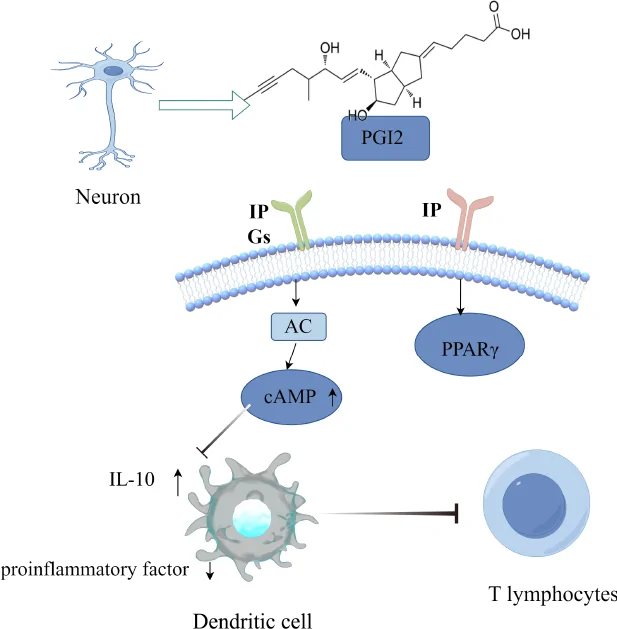
The mechanism of PGI2 immunosuppression in the nervous system. CNS-specific PGI2 is mainly expressed in neurons, exists in high concentrations in inflammatory environments, and exerts immunomodulatory effects through activation of the receptor IP. PGI2 activates IP/cAMP signaling coupled to Gs proteins, which decreases the production of a variety of pro-inflammatory cytokines and chemokines in dendritic cells and inhibits T-cell proliferation, as well as increasing the levels of the anti-inflammatory IL-10, which inhibits dendritic cell maturation and function. Dendritic cell maturation and function. It can also be mediated by the PPARγ pathway. Created with Figdraw (license code: rwSx=77777). AC: Adenylyl cyclase; cAMP: cyclic adenosine monophosphate; Gs: G protein subunit s; PPARγ: peroxisome proliferator-activated receptor gamma; PGI2: prostaglandin I2; IP: prostaglandin I2 receptor; IL-10: interleukin-10.

PGI2, also known as prostacyclin, is produced by the conversion of PGH2 in a reaction catalyzed by PGI synthase, and it belongs to the cytochrome P450 family of enzymes (Seo and Oh, 2017). PGI2 is rapidly produced following tissue injury or inflammation and is present in high concentrations in the inflammatory milieu (Bombardieri et al., 1981). PGI2 stimulates the activation of the GPCR IP to exert various biological functions. In addition to activating IP/cAMP signaling (coupled to Gs proteins), IP may be involved in the peroxisome proliferator-activated receptor γ pathway (Huang et al., 2007), and is rapidly converted to the inactive hydrolysate 6-keto-PGF1α by a non-enzymatic process that acts locally (Wu and Liou, 2005; **Figure 5**). IP can mediate injurious pain during acute inflammation, and the *IP* mRNA is present in dorsal root ganglion neurons, including those expressing substance P, which is a marker for injurious sensory neurons (Oida et al., 1995). Moreover, IP is also expressed in the spinal cord and are associated with spinal pain transmission (Doi et al., 2002; Khera et al., 2007). Khera et al. (2007) showed that the IP is located on sensory neurons and the dorsal root ganglion and dorsal root of the spinal cord, which has a high density of [^3^H]-iloprost binding sites. Intrathecal injection of carrageenan significantly induced the expression of *IP* mRNA in the spinal cord, which peaked at 6 hours, as well as COX-2 (Doi et al., 2002). In contrast, intrathecal injection of the IP agonist cicaprost induced mechanical nociceptive hypersensitivity (Doi et al., 2002).

PGI2 is a key regulator of the cardiovascular system that functions by dilating vascular smooth muscle and inhibiting platelet aggregation. Central nervous system-specific PGI2 receptors are expressed predominantly in neurons. PGI2 may be useful for the treatment of neuronal damage. This idea was confirmed by Satoh et al. (1999), who found that PGI2 protected hypoxic hippocampal neurons. However, the protective effect was not dependent on IP activation. The results of another study showed that vascular endothelium-derived PGI2 promotes neurite elongation in corticospinal neurons through cAMP signaling, and neovascular-derived PGI2 was found to promote axonal remodeling in injured neuronal networks following central nervous system inflammation (Muramatsu et al., 2012). In addition, PGI2 and its analogues have been shown to significantly reduce the amounts of various pro-inflammatory cytokines and chemokines produced by dendritic cells through the IP signaling pathway, inhibit dendritic cell-induced T-cell proliferation and cytokine production, and increase the production of the anti-inflammatory cytokine IL-10, which appeared to be related to the elevated levels of cAMP in the IP signaling pathway (Raychaudhuri et al., 2002). PGI2 and its analogues have also been demonstrated to increase during the maturation and functional inhibition of dendritic cells (Kuo et al., 2012). These findings suggest that PGI2 and its analogues play key roles in the regulation of the immune response.

### Thromboxane A2

**Figure 6:**
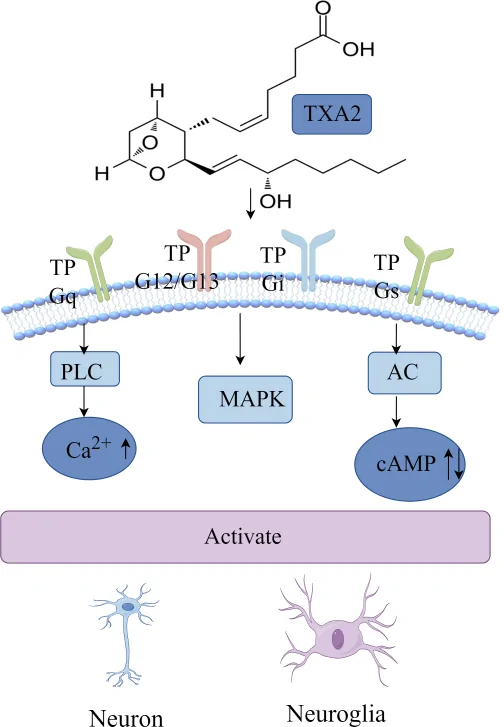
The mechanism of TXA2 immunosuppression in the nervous system. Activated microglia are the main source of TXA2 in the nervous system. TXA2 immunomodulates the nervous system by interacting with TP. TP activates Gq, G12/G13, and Gi/Gs proteins to regulate a variety of effectors such as PKC, Rho, GEF, and cAMP. TXA2 promotes the activation of cortical neurons and glial cells and exerts neuroprotective effects. Created with Figdraw (license code: rqzxw1151c). AC: Adenylyl cyclase; cAMP: cyclic adenosine monophosphate; G12/13: G protein subunit 12/13; Gq: G protein subunit q; Gs: G protein subunit s; Gi: G protein subunit i; MAPK: mitogen-activated protein kinase; PLC: phospholipase C; TP: thromboxane A2 receptor; TXA2: thromboxane A2.

TXA2, an unstable arachidonic acid metabolite, induces platelet activation and aggregation and is rapidly converted to inactive TXB2. TXA2 is formed from PGH2 catalyzed by TXS. TXA2 activity is mediated mainly by TP. Human TP has two splice isoforms, TPα and TPβ, which communicate with and heterodimerize different G proteins, leading to changes in intracellular traffic and receptor protein conformation. TP regulates a variety of effectors such as PKC, Rho, GEF, and cAMP through the activation of Gq, G12/G13, and Gi/Gs proteins (**Figure 6**).

To date, it has been determined that the role of TXA2 in neurons is limited to cellular protection against neurotoxicity. TXA2 promotes synapse growth in cortical neurons through mitogen-activated protein kinase activation (Sumimoto et al., 2015). The expression of TXA2 released as a result of ischemia can be increased in other immune-competent cells of the nervous system, including activated neuroglia, and the results of Giulian et al. (1996) demonstrated that activated microglia are the main source of thromboxane in the central nervous system. In addition, TXA2 receptors are widely distributed in different cell types and organ systems, including the nervous system (Wacker et al., 2012; Yan et al., 2024; Yang et al., 2025). After cerebral ischemia-reperfusion injury, the expression of TXA2 receptor in ipsilateral brain tissue significantly increased and was co-localized with activated microglia/macrophages in the infarcted area. This suggests that the expression of TXA2 receptor changes during pathological changes in the nervous system, implying that TXA2 may play a role in immune regulation of the nervous system (Yu et al., 2014; Yan et al., 2016). TXA2 stabilizing analogues U46619 inhibit T cell activation, and this inhibition is almost entirely dependent on ARHGEF1 (a guanine nucleotide exchange factor). In addition, platelet-derived TXA2 can inhibit T cell immunity to metastasis by inducing the immunosuppressive function of ARHGEF1 (Yang et al., 2025). This suggests that TXA2 plays a regulatory role in T cell function in the immune system. Although this study is not directly aimed at nervous system immunity, considering the integrity of the immune system and the potential role of T cells in neuroimmunity, it can also indirectly indicate that TXA2 may be involved in nervous system immune regulation. More research evidence suggests, in models of cerebral ischemia-reperfusion injury, the TXA2 receptor antagonist SQ29548 can inhibit the activation and aggregation of microglia/macrophages, including M1 and M2 phenotypes, while also reducing the upregulation of ischemia-induced IL-1β, IL-6, and TNF-α, as well as the release of inducible nitric oxide synthase (Yu et al., 2014; Yan et al., 2016; Sharif, 2023). This suggests that TXA2 may participate in the regulation of neuroinflammatory responses through its receptors, promoting the activation of microglia/macrophages and the release of inflammatory factors.

However, the different types of PGs do not act alone, and when two or more PGs are present together, they act synergistically or antagonistically on the immunomodulation of the nervous system. This depends on the different disease settings as well as the PG receptors. The PG system is involved in the pathophysiological process of many neurological diseases by regulating neuroinflammation, neuroprotection and neurodegeneration. In terms of neuroinflammation, PGs, especially PGE2 and PGI2, act as inflammatory mediators and participate in the initiation and maintenance of inflammatory response. They activate the corresponding receptors (EP1–4 and IP), promoting vasodilation, increasing vascular permeability, and recruiting immune cells into the central nervous system. In diseases such as stroke, traumatic brain injury (TBI), and MS, the overproduction of PGs exacerbates inflammatory damage, leading to neuronal dysfunction and death. In terms of neuroprotection, certain PGs or their analogs have neuroprotective effects. For example, PGE2 and its derivative misoprostol are thought to exert neuroprotective effects by inhibiting inflammatory responses, reducing excitotoxicity, and promoting neuronal survival. Additionally, PGs can regulate neurotransmitter release, synaptic plasticity, and neurogenesis, thereby facilitating the recovery of neural function. In neurodegenerative diseases, the dysregulation of PGs is closely associated with the pathogenesis of conditions such as AD, PD, and ALS. In AD, PGs may be involved in the production and clearance of Aβ, as well as the excessive phosphorylation of tau proteins, thereby promoting neuronal death and cognitive dysfunction. In PD, PGs may contribute to the selective death of dopaminergic neurons. In ALS, PGs may be involved in the degeneration of motor neurons.

## Prostaglandins and Autoimmune Neurological Diseases

Autoimmune neurological disorders are conditions wherein the body’s immune system mistakenly attacks its own neural tissues, resulting in varying degrees of neurological dysfunction. Typical examples of such diseases include MS, MG, and autoimmune encephalomyelitis. (McCombe and Pittock, 2022; Ramanathan et al., 2023). Among these, MS is the most prevalent autoimmune disease of the central nervous system. MS mainly affects young adults, and is characterized by multifocal inflammation, demyelination, and axonal damage in the central nervous system (McFarland and Martin, 2007). Patients with MS show a variety of symptoms, including visual impairment, limb weakness, paresthesia, balance impairments, and cognitive dysfunction (Marcus, 2022). The pathogenesis of MS involves interactions between genetic susceptibility and environmental factors, wherein the immune system misidentifies myelin components such as myelin basic protein as foreign antigens, activating T lymphocytes and triggering an inflammatory response to cause myelin destruction. Interestingly, the incidence of MS is significantly higher in women than in men. In the PG-mediated immune response, sex hormones may affect the expression of PG synthetase or PG receptors, leading to differences in the immunomodulatory functions of PG between men and women. One study showed that males had higher levels of PGs, likely due to increased COX-2 expression and higher nuclear factor-κB activation, than females. Additionally, males produced less leukotriene, leading to a PG diversion phenomenon (Pace et al., 2017). MG is an autoimmune nerve-muscle adapter transmission disorder (Granit et al., 2023). PGE2 is an important regulator of B-cell activation and differentiation, and it can shape the immune response by stimulating B lymphocytes to produce IgE antibodies (Fedyk and Phipps, 1996). One possible reason for the high incidence of MG in women is that female immune cells are more sensitive to PG. A study showed that mononuclear cells isolated from women, when activated *in vitro* with LPS, produced more PG than male mononuclear cells (Leslie and Dubey, 1994). Patients with MG show postsynaptic membrane acetylcholine receptor autoantibodies at the nerve-muscle joint, and the combination of these antibodies with the acetylcholine receptor hinder the normal combination of acetylcholine and the receptor, affecting the transmission of nerve impulses and leading to muscle weakness. The symptoms of MG are usually characterized by lightness in the morning and heaviness in the evening, and can involve the ocular muscles, the muscles required for chewing, and the throat muscles; these symptoms can also affect respiratory muscles, potentially making them life-threatening (Iorio, 2024). However, determination of the etiology and pathogenesis of MS *in vitro* and *in vivo* is very difficult. Recent lipidomics studies have provided a new perspective on the spectrum of PG metabolites in diseases. Lipidomics analysis of cerebrospinal fluid from patients with MS showed that the levels of various PG metabolites, including PGE2 and PGF2α, were significantly elevated, and these levels were associated with the disease’s recurrence and remission cycles. Notably, an abnormal increase in the levels of PGE2 metabolites was positively correlated with the degree of inflammatory cell infiltration, suggesting that it may serve as a potential biomarker for disease activity (Lopez-Pier et al., 2018). Although direct lipidomics studies on MG are lacking at present, future studies could employ the research approaches used in other autoimmune diseases to analyze the profiles of PG metabolites in patients’ serum or saliva to identify metabolite characteristics related to anti-acetylcholine receptor antibody levels, providing new directions for early diagnosis and disease monitoring. Autoimmune encephalitis is a type of encephalitis mediated by autoimmune mechanisms that can be caused by anti-neuronal cell surface antigen antibodies or anti-neuronal intracellular antigen antibodies (Bastiaansen et al., 2021). The clinical manifestations of autoimmune encephalitis are complex, and include mental behavior abnormalities, cognitive impairment, seizures, speech disorders, and motor disorders. For example, anti-NMDA receptor encephalitis is often common in young women and children, and is often accompanied by ovarian teratoma and other tumors; the pathogenesis of this condition is related to the fact that anti-NMDA receptor antibodies interfere with the normal functioning of neurons (Dalmau and Graus, 2023; Nguyen and Wang, 2023). Despite differences in the specific pathogenic mechanisms of different autoimmune neurological diseases, all of these diseases involve overactivation of the immune system and disruption of self-tolerance.

PGE2 regulates the immune response by combining with the EP receptors (EP1–4) on the surface on the surface of immune cells. It inhibits the differentiation of Th1 and Th17 cells, which secrete cytokines that play a key role in the autoimmune inflammatory response, such as IFN-γ and IL-17. At the same time, PGE2 also promotes the generation of Tregs, whose immunosuppressive functions can maintain the immune balance and reduce the autoimmune damage to the nervous system (Bettelli et al., 2006, 2008; Kroenke et al., 2008). Elevated expression of PGE2 in autoimmune neurological disorders, such as MS (Mattsson et al., 2009) and experimental autoimmune encephalomyelitis (EAE) (Esaki et al., 2010), exhibits a marked immunosuppressive effect on these diseases. PGE2 has been shown to contribute to the suppression of mesenchymal stem cell-mediated non-specific T-cell proliferation *in vitro* and antigen-specific T-cell proliferation in MS patients (Zafranskaya et al., 2013). A recent study using T cells deficient in each EP subtype found that the immunosuppressive effects of PGE2 were mediated by EP2 and EP4 (Nataraj et al., 2001). In an *in vitro* experiment, the receptors for PGE2, EP2 and EP4, synergized with related cytokines to promote Th1 cell differentiation and Th17 cell expansion, and the administration of EP4 antagonists attenuated EAE development *in vivo* (Yao et al., 2009). Another promising therapeutic intervention is the reduction of demyelination and motor dysfunction in a mouse model of copper-based toxin-induced MS by AL-8810 (PGF2α receptor FP inhibitor), which was mediated by a reduction in astrocyte proliferation (Iwasa et al., 2014). In autoimmune encephalitis, PGD_2_ can attract eosinophils, Th2 cells, and other cells to migrate to the inflammatory site. The cytokines secreted by Th2 cells, such as IL-4 and IL-5, can modulate the immune response to balance Th1 and Th17 cells. Th2 cell hyperactivation may also lead to immune imbalance and aggravate disease symptoms (Kupczyk and Kuna, 2017; Chen et al., 2024). PGE₂ may regulate the balance of T-cell subsets, affect the secretion of cytokines, and subsequently affect autoantibody production against the acetylcholine receptor. However, the specific mechanism has not been fully defined. PGs may also affect neurotransmitter release or the sensitivity of the postsynaptic membrane (Mirshafiey and Jadidi-Niaragh, 2010). Although the specific role of PGs in MG remains unclear, this potential impact provides a new direction to further investigate the pathogenesis of MG, which still needs to be confirmed by more studies. These studies highlight the complex and critical role of PGs in autoimmune neurological diseases. Since PGs are not only inflammatory mediators, but are also involved in the regulation of immune homeostasis in the nervous system, they can be expected to show important effects on the development and outcome of autoimmune neurological diseases.

## Prostaglandins and Cerebrovascular Destructive Disease

Cerebrovascular destructive diseases are a group of diseases presenting with impaired cerebrovascular structure and function that subsequently induces brain tissue damage and neurological dysfunction. Common cerebrovascular destructive diseases include cerebral hemorrhage, cerebral infarction, subarachnoid hemorrhage, and cerebral arteriovenous malformation (Watanabe and Hashimoto, 2022; Choi et al., 2023). Severe cerebrovascular injury diseases such as stroke and head trauma/mechanical injury are usually caused by vascular abnormalities or cerebrovascular injury and are, therefore, accompanied by ischemia/hypoxia. Some PGs, such as PGI₂, have potent vasodilatory effects, which can increase intracellular cAMP levels through activation of adenylate cyclase and thereby lead to vascular smooth muscle diastole, increasing cerebral blood flow, improving blood supply to brain tissue, and potentially protecting against ischemic cerebrovascular disease (Yagami et al., 2016). Following hypoxia, PGE2, PGD2, and PGF2α exacerbate neuronal cell death. Permanent middle cerebral artery occlusion in rodents has been used as a reliable and predictive model of stroke. In this model, the key synthase COX-2 is strongly induced in neuronal cell bodies and dendrites in the peri-infarct region. Administration of selective COX-2 inhibitors (e.g., NS398 and SC58236) has been shown to reduce the infarct volume and ameliorate neurological deficits in mice and rats after experimental cerebral ischemia-reperfusion (Sugimoto and Iadecola, 2003). In contrast, COX-2-deficient mice show reduced susceptibility to ischemic brain injury and NMDA-mediated neurotoxicity. EP1 antagonists may also be beneficial in the management of stroke, since SC51089 and SC51322 have been shown to prevent damage to hippocampal slices in rats (Zhou et al., 2008). Similarly, McCullough et al. (2004) used isolated hippocampal neurons and slices placed under NMDA-induced cytotoxic and hypoxic conditions, and their findings suggested that activation of EP2 may be neuroprotective in cerebral ischemic challenge; this effect was mediated by the cAMP-PKA signaling pathway. A selective central nervous system-type IP agonist was shown to eliminate brain damage in an animal model of stroke.

TBI is a very common stroke-related brain injury and is often associated with brain damage and death in the elderly. TBI is characterized by the presence of hematomas, subarachnoid hemorrhage and diffuse damage after initial contusion, as well as axonal death of cerebral neurons. The combination of neuronal inflammation with oxidative stress and cerebral neuronal Ca^2+^ overload represents the second stage of TBI and the ensuing loss of a variety of bodily functions, including speech and neuromuscular activity. PGs have long been recognized as potent pro-inflammatory modulators, and blocking PG synthesis and activity has become a widely accepted treatment for inflammation-related diseases. However, a growing number of recent reports suggest that PGs can exert toxic and paradoxical protective effects during inflammation (Sandig et al., 2007). Several researchers have reported increased production and release of pro-inflammatory PGs, including PGF2α. Interestingly, elevated levels of COX-2 have been observed in ischemic neonatal and adult human brains, and in an experimental mouse model of TBI, Glushakov et al. (2013) also demonstrated that intraperitoneal injection of an FP antagonist (AL-8810) reduced hippocampal swelling and improved neurological deficit scores at 1 and 2 days after TBI injury. Thus, FP antagonists may be used to reduce injury and help maintain the functional integrity of the cerebral cortex and hippocampus after and during TBI events. PGs perform complex roles in cerebrovascular destructive diseases, and this complex role depends on different types of PGs and their specific receptors. Thus, the application of PG system-related enzyme inhibitors and receptor antagonists holds promise for improving neurological defects and maintaining the functional integrity of the brain.

## Prostaglandins and Neurodegenerative Diseases

Neurodegenerative diseases are a class of chronic and progressive neurological diseases characterized by progressive loss of neuronal structure and function, which seriously affect the quality of life and life span of patients and impose heavy burdens on the family and society (Heemels, 2016; Agnello and Ciaccio, 2022). Neurodegenerative diseases are usually characterized by an insidious onset, progressive development, and a prolonged disease course. At present, most of these diseases lack effective radical cure methods. Their etiology and pathogenesis are complex, involving interactions of genetic factors, environmental factors, neuroinflammation, oxidative stress, protein misfolding, and other factors (Heemels, 2016; Logroscino et al., 2022).

AD, one of the most common neurodegenerative disorders, is a degenerative disease of the central nervous system that occurs in old age and preadolescence and is characterized by progressive cognitive dysfunction and behavioral impairments, with cortical atrophy, neuronal loss, neuroprogenitor fiber tangles, senile plaques, and Aβ deposits in various regions of the brain. COX-1 has been shown to be expressed in the microglial cells surrounding the amyloid plaques of the AD brain. Neuronal COX-2 is upregulated in the pyramidal layer of hippocampal CA1–4 regions in patients with AD (Yermakova et al., 1999; Hoozemans et al., 2001). COX-2 has also been shown to be induced in the neurons of a transgenic mouse model of AD and in Aβ-treated SH-SY5Y neuroblastoma cells (Hoozemans et al., 2001). The upregulation of COX-1 and COX-2 expression can produce large amounts of PGE2, which binds to EP on the cell surface, activating downstream Gs proteins and subsequently increasing intracellular cAMP levels. cAMP can activate PKA, which acts on the transcription factor CREB. Phosphorylated and activated CREB binds to the promoter region of the Aβ precursor protein cleaving enzyme 1 (*BACE1*) gene, enhancing its transcriptional activity and leading to increased mRNA expression of BACE1. This ultimately results in elevated BACE1 protein levels, promoting the Aβ production pathway of APP and increasing the production and deposition of Aβ, thereby driving the progression of AD (Liang et al., 2005b; Bi et al., 2025). Clinical trials have reported that treatment with indometacin (a nonselective COX inhibitor) improved cognitive impairment in AD (Rogers et al., 1993), while administration of naproxen (a selective COX-2 inhibitor) blocked Aβ-mediated inhibition of long-term potentiation and memory function in Tg2576 AD model mice (Kotilinek et al., 2008). Non-steroidal anti-inflammatory drugs may reduce the risk of AD, and their beneficial effects are related to their ability to inhibit inducible COX-2 at the site of inflammation and inhibit the adverse effects of constitutive COX-1 expression (e.g., gastric injury). Montin et al. detected abnormally high levels of PGE2 and EP2-induced elevation of Aβ precursor mRNAs and proteins in the cerebrospinal fluid of patients with AD (Greco et al., 1999). Some investigators have observed PGE2 stimulation of microglial activation and widespread neuroinflammation in AD model mice (Xia et al., 2023), which appears to be mediated by EP2, with which EP3 shares features. In contrast, EP4 exerts a protective anti-inflammatory effect in AD models. A targeted metabolomics analysis using liquid chromatography-mass spectrometry on 147 plasma-cerebrospinal fluid samples from the Barcelona AD Center and 58 cerebrospinal fluid samples from patients with mild cognitive impairment in the Mannheim/Hidelberg cohort revealed that biomarkers of oxidative stress (8,12-iso-iPF2α VI) and inflammation (PGF2α) are associated with biomarkers of pre-Alzheimer’s disease pathology. The relationship between PGF2α and tau pathology markers may be influenced by the APOE genotype (Ahmad et al., 2024). Moreover, IP is also involved in the AD phenotype, with agonist-induced activation of IP stimulating the production of soluble amyloid precursor protein α with a neuroprotective subtype in isolated human microvascular endothelial cells (He et al., 2017).

PD is another common neurodegenerative disorder characterized by disabling motor abnormalities, including tremors, muscle rigidity, lack of voluntary movement, and postural instability. The main neuropathological feature of PD is the loss of neurons containing nigrostriatal dopamine, whose cell bodies are located in the substantia nigra pars compacta, striatum, and in nerve endings. During disruption of the nigrostriatal pathway, COX-2 expression in dopaminergic neurons has been shown to be specifically induced in the substantia nigra pars compacta of postmortem PD specimens and in a 1-methyl-4-phenyl-1,2,3,6-tetrahydropyridine mouse model of PD (Teismann et al., 2003). Inhibition of COX-2 by acetylsalicylic acid and salicylic acid has been shown to provide neuroprotection in the 1-methyl-4-phenyl-1,2,3,6-tetrahydropyridine model (Teismann and Ferger, 2001). Thus, COX-2 inhibitors have therapeutic potential for PD. PGA1, as a metabolite of COX-2, also plays an important role in PD-related studies. In experiments with transgenic mice (a commonly used animal model for PD), short-term administration of PGA1 was shown to significantly reduce the phosphorylation levels of tau protein at the Thr181, Ser202, and Ser404 loci in a dose-dependent manner. In-depth research studies have shown that PGA1 can undergo a Michael addition reaction with cysteine 377 on the scaffold subunit A alpha (PPP2R1A) of protein phosphatase 2A, thereby activating protein phosphatase 2A. As an important protein phosphatase, protein phosphatase 2A can dephosphorylate overphosphorylated tau protein, restoring its normal functional state and reducing the formation of neurofibrillary tangles, thus providing some protective effect against PD (Liang et al., 2005b). A recent study involving the induction of neurotoxicity by 6-hydroxydopamine via oxidative stress in a neuroblastoma cell line (SH-SY5Y) demonstrated the protective effects of FP agonists, fluprostenol, and PGF2α (Zhu et al., 2021). Although the EP receptor agonist butatoprost has been shown to protect isolated dopaminergic neurons in cultures (Prinzmetal et al., 2008), its efficacy in patients with PD remains to be determined.

ALS is a chronic progressive neurodegenerative disease characterized by invasion of both upper and lower motor neurons. This condition causes progressive paresis and muscle atrophy, but no effective treatment exists. As a result, patients with ALS usually die within 1–5 years of disease onset (Bruijn et al., 2004). Existing studies have shown that inflammation and immune responses play important roles in the pathogenesis of ALS, and PG as an inflammatory mediator may play a role in the neuroinflammatory process of ALS, as reflected by the elevated levels of proteins associated with inflammatory states, such as PLA2, COX-1, and COX-2 (McGeer and McGeer, 2002). Furthermore, PGE2 levels have been shown to be elevated in postmortem brain tissue, cerebrospinal fluid, and serum samples of patients with sporadic ALS (Iłzecka, 2003). EP receptors have also been shown to be involved in accelerating disease progression in animal models of ALS. PG works by binding to EP2 on the cell surface, which is activated to cause changes in intracellular cAMP levels that affect downstream signaling pathways (Sahu et al., 2024). PGs may exacerbate neuroinflammation by activating the Toll-like receptor 4 signaling pathway, thereby promoting the progression of ALS (Winter and Bickford, 2019). Thus, antagonism of EP2 may contribute to slowing the progression of ALS. Inhibition of PGE2 biosynthesis using COX-2 inhibitors has also yielded beneficial therapeutic effects in ALS model mice, although the usefulness of these inhibitors has not been demonstrated in clinical trials (Nango et al., 2023).

MS is a debilitating chronic inflammatory condition affecting the central nervous system, and is characterized by persistent inflammation, demyelination, gliosis, varying levels of axonal and oligodendrocyte damage, progressive neurological impairment, and significant infiltration by a diverse array of immune system cellular and soluble mediators (Mirshafiey and Jadidi-Niaragh, 2010). However, both *in vitro* and *in vivo* analyses of the etiology and pathogenesis of MS are difficult, and only some progress has been made in developing MS rodent models using EAE, in which PG plays a very important role. The PG synthesis enzymes PLA2 and COX, particularly COX-2 and mPGES-1, exhibit elevated expression levels in the lesion sites associated with MS, thereby facilitating the generation of PGE2 and subsequently contributing to neuroinflammation and demyelination (Mirshafiey and Jadidi-Niaragh, 2010; Palumbo et al., 2012; Gulla et al., 2020). Inhibitors of COX-1/2 and PLA2 have been shown to reduce the symptoms and behavioral effects associated with EAE (Malmberg et al., 2004). One additional promising therapeutic intervention pertains to the use of an FP-receptor antagonist (Sharif et al., 2000) in reducing myelin loss and movement disorder in a mouse model of MS using copper-based toxin (Iwasa et al., 2014). Thus, pharmacological agents targeting COX-2 and mPGES-1 may offer promising therapeutic avenues for the management of MS. Xu et al. (2013) demonstrated that the cooperation between PGE2 and adenosine can suppress the progression of EAE. They observed that the combination of PGE2 and adenosine significantly reduced the production of INF-γ and IL-17 from T cells. This inhibitory effect was mediated through EP4 and A2A receptors. PGE2 increases Th1 cell differentiation and Th17 cell expansion through the EP2/EP4 signaling pathway, thereby exacerbating neuroinflammation (Liu et al., 2013). Therefore, the use of EP2/EP4 antagonists may help inhibit the development of MS. However, a contrasting study showed that EP4 agonists have neuroprotective and anti-inflammatory effects in animal models of MS. They can reduce inflammatory cell infiltration in the central nervous system, reduce blood–brain barrier permeability, inhibit axonal damage, and promote myelination regeneration (Esaki et al., 2010; Schiffmann et al., 2014). These findings highlight the dual roles of pro-inflammatory and immunomodulatory abilities depending on the environment and interactions with other mediators.

Huntington’s disease (HD) is an autosomal dominant pathological disease caused by the abnormal expansion of CAG repeats in the Huntington gene (*HTT*) located on chromosome 4p16. This disease is characterized by movement disorders and dementia (Bates et al., 2015; Tong et al., 2024). The debilitation associated with HD, including movement disorders and dementia, appears to have some linkage to PGs and inflammation, since administration of COX inhibitors in animal models of HD helped reduce some of these symptoms. PGE2 acts through its receptor (EP receptor). The EP receptor belongs to the superfamily of GPCRs, and studies have shown that GPCRs can indirectly affect the signal pathway of brain-derived neurotrophic factor/tyrosine kinase receptor B (TrkB), which is crucial for neuronal survival (Antonijevic et al., 2025). Therefore, PG may affect the neurodegenerative process of HD by regulating GPCRs and affecting the brain-derived neurotrophic factor/TrkB signaling pathway. Besides, one study showed that a PGE2 EP1 antagonist improved motor deficits and rescued memory decline in an R6/1 mouse model of HD (Anglada-Huguet et al., 2014). Misoprostol (an EP2/EP3 agonist) also showed a neuroprotective role in reducing memory reduction in the R6/1 mouse model of HD through the release of brain-derived neurotrophic factor (Anglada-Huguet et al., 2016). However, other types of PGs have been relatively less well studied in HD.

To date, accumulating evidence has demonstrated that PGs can have opposite effects on neurodegenerative diseases, either contributing to neuroprotection or enhancing neuroinflammatory and neurodegenerative processes. The complex and critical role of PGs depends on the different types of PGs and their specific receptors, providing key targets and ideas for related research and therapeutic strategies.

## Prostaglandins and Neuralgia and Chronic Pain

Neuropathic pain refers to the pain produced by the nervous system itself in the absence of external stimulation. This form of pain is usually characterized by severe intensity, diverse nature, and irregular attack times (Bannister et al., 2020). In the peripheral mechanism underlying neuropathic pain, when the tissue is stimulated by injury or inflammation, the damaged cells release a variety of chemicals, such as PGs, bradykinin, and histamine. These substances can activate nociceptors on peripheral nerve terminals to produce nerve impulses. Simultaneously, the damaged nerve fibers may undergo ectopic discharge, thereby spontaneously producing impulses in the absence of normal stimulation, and these abnormal impulses are afferent to the central nervous system, which will produce pain sensation. Moreover, the expression and function of sodium and calcium channels are altered after nerve injury, increasing the excitability of nerve fibers and allowing easier generation and conduction of pain signals (Devor et al., 1989; Cohen et al., 2021; Brackx et al., 2023). Furthermore, the central mechanism involves peripheral nerve impulses afferent into the spinal cord and information processing and transmission in the dorsal horn of the spinal cord. The excitability of spinal dorsal horn neurons can be altered to cause pain sensitization, which manifests as a pain response to normal non-nociceptive stimuli or an enhanced pain response to nociceptive stimuli. The cerebral cortex also plays a very important role for the perception and processing of pain. When the nerve impulse is transmitted to the cerebral cortex, the brain will locate the pain and evaluate the intensity and nature of the pain. In addition, emotional, cognitive and other regions in the brain also interact with pain-processing regions to influence an individual’s subjective feelings and emotional responses to pain (Cohen and Mao, 2014).

PG is associated with the mediation of neuroinflammation and pain, which led to the discovery of aspirin, a frequently used COX-1 inhibitor that serves as an analgesic. The hypothalamus has been shown to regulate skeletal remodeling and structure in response to mechanical loading by sensing the concentration of PGE2 in response to mechanical loading, and this mechanism is associated with pain. In the gray matter of the midbrain aqueduct, PGs inhibit the release of the excitatory neurotransmitter glutamate by acting on EP3 at the presynaptic nerve terminal, weaken the descending inhibition of pain, and thereby strengthen supraspinal injury stimulation, leading to central sensitization and enhanced pain perception (Lu et al., 2007). In cases involving tissue injury and inflammation, locally released PGs can increase the sensitivity of peripheral nerve endings, reducing the pain threshold such that stimuli that normally do not cause pain generate pain signals. This process is known as peripheral sensitization. For example, in arthritis models, injecting PGs into the joint cavity can lead to increased sensitivity of the nerves around the joint to mechanical and thermal stimuli (Ohashi et al., 2023). PGs can also regulate the functioning of immune cells and promote the release of inflammatory cytokines such as IL-1 and TNF-α, which can further promote the synthesis and release of PGs, forming a vicious circle of inflammation and pain. Moreover, PGE2 has been shown to sensitize the TRPV1 capsaicin channel through EP1 and IP (Aihara et al., 2005). DP1 and EP2 mediate inflammatory nociceptive sensitization in the spinal cord, and EP3 and κ-opioid receptors co-operate in transducing tactile pain (Minami et al., 2003; Reinold et al., 2005; Grill et al., 2008). In addition, the FP antagonist AL-8810 reduced intrathecal Aβ-methylene adenosine triphosphate-induced aberrant pain in wild-type mice (Wu et al., 2023). Overall, various PG receptors are actively involved in mediating different forms of nociceptive signaling.

## Clinical Studies on the Use of Prostaglandins for Neurodegenerative Diseases

As mentioned above, neurological diseases such as neurodegenerative diseases and ischemic brain injury pose a serious threat to human health, and effective radical cure methods are currently unavailable for most of these diseases. Although PG drugs show potential for the treatment of neurological diseases, research on these drugs is associated with many challenges. As early as 1999, investigators conducted a double-blind, placebo-controlled trial of diclofenac/misoprostol for AD, and although the trial did not demonstrate a significant effect of non-steroidal anti-inflammatory drug treatment on AD, the observed trends highlighted the need for further research with more participants (Scharf et al., 1999). Subsequent studies investigating the effects of rofecoxib and naproxen with placebo on AD progression also did not demonstrate that PGs could slow cognitive decline in patients with mild-to-moderate AD (Aisen et al., 2003; Reines et al., 2004). A trial using celecoxib for ALS yielded the same results (Cudkowicz et al., 2006). Although celecoxib may have little effect on neurodegenerative diseases, it may play a role in neuralgia. Tolfenamic acid is a non-steroidal anti-inflammatory drug that reduces PG synthesis by inhibiting the activity of COX (Xu et al., 2022a). In a double-blind crossover study, 160 patients took tolfenamic acid (a potent inhibitor of PG biosynthesis), tartrate, acetylsalicylic acid, or placebo during migraine attacks (SciNeuro Pharmaceuticals, 2023). Preliminary results from a randomized, double-blind, placebo-controlled, single- and multiple-dose-escalation phase I clinical study of the lipoprotein-associated PLA2 inhibitor Rilapladib (SB659032) in healthy individuals showed that SB659032 was safe and well-tolerated and showed no serious adverse events; pharmacokinetic and pharmacodynamic data showed that the product had good exposure in the body after oral administration, supporting once-daily administration and good distribution in cerebrospinal fluid, and could completely inhibit the target enzyme activity in the peripheral and central nervous system (Maher-Edwards et al., 2015). Tolfenamic acid and ergotamine have been shown to be equally effective in reducing the duration and intensity of episodes, but the adverse effects of tolfenamic acid, especially nausea, are less common (Hakkarainen et al., 1979). The effectiveness of tolfenamic acid in acute migraine attacks is consistent with the role of PGs in migraine (Hakkarainen et al., 1979). Limaprost, a synthetic derivative of PGE, is the first drug in China to be developed based on research into the pathological mechanism of lumbar spinal stenosis. It can improve microcirculatory disorders in neural blood flow and promote the recovery of neural function (Kim et al., 2016; Park et al., 2024). In addition, a 12-week preclinical study on tolfenamic acid treatment in patients with progressive supranuclear palsy (a rare, progressive neurodegenerative disease mainly involving the midbrain, pons, vertical ophthalmoplegia, Parkinson’s syndrome, pseudobulbar paralysis, and cognitive dysfunction as the main clinical features) is ongoing (NCT04253132). Although the results of the study have not yet been released, the findings may yield a breakthrough for progressive supranuclear palsy and offer hope for tackling similar diseases. Diclofenac, rolofecoxib, and celecoxib all target COX-2, which may be responsible for their poor treatment efficacy for neurodegenerative diseases.

Although many clinical trials have shown great promise for the use of PGs and their analogs in the treatment of neurological diseases, many limitations remain unresolved. Many failed clinical trials, such as those using diclofenac, rofecoxib, and celecoxib, have focused on selective inhibition of COX-2 (Fine, 2002; Hochberg, 2005). COX-1 and COX-2 perform different roles in the nervous system. COX-1 may be involved in maintaining cerebral blood flow and early inflammatory responses (Pluchino and Alfaro-Cervello, 2011; Mishra, 2017). However, some findings suggest that excessive inhibition of COX-1 may disrupt cerebral blood flow homeostasis, which, in turn, affects brain tissue status (Niwa et al., 2001). COX-2 overexpression is one of the hallmarks and effectors of neurological damage after brain injury, and COX-2 inhibitors can exert neuroprotective effects by reducing PG and free radical synthesis. However, Strauss (2008) demonstrated that over-inhibition of COX-2 may interfere with certain endogenous neuroprotective mechanisms *in vivo*, which is detrimental to neuroprotection. They also found that COX-2 plays a dominant role in arachidonic acid metabolism, and that excessive inhibition of COX-2 may lead to the diversion of arachidonic acid to other metabolic pathways, affecting the overall homeostasis of PGs and their neurophysiological functions. Therefore, excessive selective inhibition of COX-2 may interfere with the overall balance of PGs, which is not conducive to neuroprotection. Clinical trials have often paid insufficient attention to specific PG pathways, which are diverse and functionally different. Clinical trials usually focus only on the inhibition of COX enzymes, while ignoring the regulation of specific PG receptors or downstream signaling pathways. Future research should focus more on specific types of PGs, such as PGE1 and its derivative limaprostin in improving neurovascular microcirculation, or the potential of PGF2α in clearing brain waste. Therefore, identification of more PG analogues or more downstream target drugs may hold greater promise. In addition, neurodegenerative diseases such as AD have a long disease course, and their pathogenesis and treatment needs may differ across stages. As a result, including patients with both mild and moderate disease in the same trial may mask the efficacy of drugs at specific stages. The failure of celecoxib to treat ALS may reflect the complexity of the pathogenesis of ALS, and inhibition of COX-2 alone may not be effective in interfering with disease progression. We emphasize that combinations of PG system targets with other therapeutic agents and modalities, such as immunomodulatory drugs and gene therapy, may yield better results.

## Research Outlook and Conclusion

In neuroscience, substantial progress has been made in the study of PGs. The identification of key enzymes involved in PG biosynthesis, degradation, and biological functions has helped characterize the molecular basis for PG production. Additionally, microbial metabolites and epigenetic mechanisms have also been shown to influence PG biosynthesis. The diverse biological functions and unique immunosuppressive effects of PGs provide important insights into the physiological and pathological mechanisms of the nervous system. PGs can exert immunosuppressive effects through multiple pathways. These include modulating the functions of neuronal cells (especially neurons and glial cells), affecting the body’s immune response. PGs can act as an immunosuppressors by inhibiting the release of inflammatory mediators and the proliferative activation of cells, reducing the inflammatory response. In addition, PGs can also influence the functioning of immune cells by activating intracellular signaling pathways through binding to their specific receptors, regulating cytokine production and cytotoxicity in immune cells. These mechanisms are crucial for autoimmune neurological diseases, neurodegenerative diseases, and neuroinflammation such as neuralgia, and favorable therapeutic results have been achieved in the treatment of a number of disorders by utilizing the immunosuppressive function of PGs. However, the immunosuppressive effects of the PG system can also have undesirable consequences. In some cases, excessive immunosuppression may lead to increased susceptibility to infections and other diseases, which has limited the clinical use of PGs.

Although these findings have laid a solid foundation for exploration of PGs in neuroscience, the existing reviews and other related studies still have multidimensional limitations. In terms of literature citation and research content, more than 70% of the studies rely on rodent models and *in vitro* cell culture systems. Considering AD research as an example, while rodent models can simulate pathological features such as Aβ deposition through gene editing, they differ substantially from humans in terms of neural circuit complexity, lifespan, and gene expression regulation (Qian et al., 2024; Yeapuri et al., 2025). The human brain contains approximately 86 billion neurons, whereas the mouse brain has only about 70 million neurons. This significant difference in neuron numbers implies that the complexity of neural signal transmission networks in animals such as mice cannot be equated to that in humans (Williams, 2000). While *in vitro* cell experiments can accurately control variables, they cannot easily replicate various elements of the complex microenvironment of the nervous system, such as the blood–brain barrier and glial cell network. Moreover, the metabolic coupling of astrocytes and neurons cannot be completely presented in the cultured neurons *in vitro*, limiting the universality of research conclusions in clinical transformation.

Furthermore, PGs include various active products such as PGE2, PGI2, PGD2, PGF2α, and TXA2. These subtypes exhibit overlapping and antagonistic functions in the nervous system. For example, PGE2 can activate inflammatory responses by activating inflammasomes during the early stages of acute brain injury. However, during the repair phase, it plays a neuroprotective role by promoting the secretion of neurotrophic factors. The molecular switch mechanism that regulates these dual functions remains to be fully understood. This article provides a comprehensive overview of the process by which PG regulates various cells associated with the nervous system, thereby further regulating neurological diseases. However, PG also interacts extensively with neurotransmitters (such as dopamine and serotonin), cytokines (IL-1β and TNF-α), and ion channels in signaling pathways. The relationship between these factors and PG, and whether they can interact to influence the onset and progression of neurological diseases, has not been thoroughly explored in this article. However, studies have shown that human umbilical cord mesenchymal stem cells can alleviate neuroinflammation, inhibit the activation of microglia in the central nervous system, and increase the levels of monoamine neurotransmitters like serotonin and brain-derived neurotrophic factor. These effects can help alleviate depressive and anxious behaviors, indicating a link between neuroinflammation and monoamine neurotransmitters (Liu et al., 2025). Furthermore, literature indicates that neuroinflammation plays a crucial role in the pathogenesis of various neurological disorders. Central nervous system cells can produce prostaglandins, which can exert their effects by activating known receptors or through non-classical mechanisms. For instance, PGE2 can modulate the expression of inflammatory mediators in microglia, providing a theoretical basis for the synergistic regulation of neuroinflammation by PGs and neurotransmitters (Lima et al., 2012). Therefore, the synergistic effects of PGs and neurotransmitters may become a new hub for the regulation of neuroinflammation.

In the realm of disease transformation and research ethics, studies related to PGs face substantial challenges. Most of the existing studies have focused on exploring fundamental mechanisms, while preclinical translational studies for major neurological diseases such as AD, PD, ischemic stroke, and epilepsy are currently lacking. The acquisition of human brain tissue samples is subject to strict ethical regulations, and patients with neurological disorders show notable differences in genetic characteristics, disease progression, and treatment history, presenting challenges in the design of large-scale, standardized clinical cohort studies. Therefore, our article presents relatively little literature on clinical research. Moreover, due to the limitations of detection technologies, real-time, high-resolution spatiotemporal monitoring of PGs within nerve cells is challenging. Traditional immunohistochemistry and enzyme-linked immunosorbent assay techniques can only provide end-point content data, and they cannot identify the instantaneous changes in PGs during the brief onset of neuroinflammation (lasting about a few minutes). Additionally, data integrating the metabolite profiles of PGs are currently lacking. Future studies should aim to integrate cutting-edge technologies such as single-cell sequencing, spatial transcriptomics, gene-edited animal models, and high-resolution imaging to systematically analyze the regulatory network of the PG system in both neurophysiological and pathological conditions. Additionally, efforts should be intensified to conduct multi-center clinical studies, establishing the relationship between PG-related biomarkers and disease diagnosis and prognosis, thereby accelerating the transition from basic research to clinical application.

In addition, this review has some limitations in terms of research design. First of all, there is still a large space for expansion in the depth and breadth of research direction. Current research on prostaglandins PG gistic or antagonistic effects between PG and other neurotransmitters (such as dopamine and 5-hydroxytryptamine) and neuroregulators (such as neuropeptides and growth factors). PGs have both pro-inflammatory and anti-inflammatory properties in the inflammatory process, and their mechanism of action is complex. However, we did not discuss the pro-inflammatory and anti-inflammatory properties of prostaglandins in different sections, which led to a superficial explanation of this part.

This review provides new ideas and approaches for the use of PGs in the treatment of diseases. In-depth future studies on the immunosuppressive effects of the PG system may yield more biomarkers and therapeutic targets related to PGs. In this regard, PG analogues and their downstream targets that may play a greater therapeutic role with fewer adverse reactions should receive more attention. The specific mechanisms of PGs in neuroimmunomodulation, including the interactions between different types of PGs, interactions between different types of PGs and various neurotransmitters, and their roles in neuropathological conditions, should also be investigated in more detail. These findings can help researchers identify potentially novel therapeutic strategies and prophylactic measures for better patient recovery.

## Data Availability

*Not applicable.*
